# Effectiveness of a standard clinical training program in new graduate nurses’ competencies in Vietnam: A quasi-experimental longitudinal study with a difference-in-differences design

**DOI:** 10.1371/journal.pone.0254238

**Published:** 2021-07-09

**Authors:** Satoko Horii, Chinh Thi Minh Nguyen, Huong Thi Thu Pham, Naomi Amaike, Hien Thi Ho, Hirotsugu Aiga

**Affiliations:** 1 Center for Next Generation of Community Health, Chiba University Hospital, Chiba, Japan; 2 Post Graduate Training Department, Nam Dinh University of Nursing, Nam Định, Vietnam; 3 Faculty of Nursing, Phenikaa University, Hanoi, Vietnam; 4 JICA Project for Strengthening Clinical Training System for New-Graduate Nurses, Vietnam; 5 Faculty of Clinical Medicine, Ha Noi University of Public Health, Hanoi, Vietnam; 6 School of Tropical Medicine and Global Health, Nagasaki University, Nagasaki, Japan; Hamamatsu Ika Daigaku, JAPAN

## Abstract

**Objective:**

This study aimed to estimate the effectiveness of a standard clinical training program for new graduate nurses in Vietnam.

**Methods:**

A quasi-experimental longitudinal study with a difference-in-differences design was conducted. A total of 280 new graduate nurses completed a self-administered questionnaire. The intervention group consisted of 206 respondents (those having participated in standard clinical training) and the control group (those that did not receive training) of 74. Differences in mean increases in competency scores between the intervention and control groups were estimated. The effect size of the intervention was estimated by calculating Cohen’s d. A generalized linear model was employed to identify the factors associated with mean increases.

**Results:**

The mean increase in total competency scores (range: 0–6 points) in the intervention group was 0.73 points greater than in the control group with an intermediate effect size (Cohen’s d = 0.53; 95% CI 0.26 to 0.80). A greater reduction in standard deviation of total competency scores in the intervention group was confirmed. Participation in standard clinical training produced a positive association with a mean increase in total competency score without significance (β = 0.04, P = 0.321). Provincial hospitals as clinical training venues had a significantly positive association (β = 0.11, P = 0.007) with the mean increase in total competency scores. Competency at pre-clinical training was negatively (β = -0.75, P < 0.001) associated with the mean increase.

**Conclusion:**

Findings implied that the standard clinical training program could contribute to both increasing and standardizing new graduate nurses’ competencies in Vietnam. Further studies are needed to more precisely examine the attribution of standard clinical training to better increase new graduate nurses’ competencies.

## Introduction

People’s health problems and their need for health services are becoming more diverse and complex than ever due to epidemiologic, socio-demographic, and socio-economic changes, which have increased the importance of ensuring patient safety and patient-centered care. To adequately respond to patients’ diverse demands, nurses are required to be equipped with higher competencies, regardless of the types of countries and health facilities in which they work [1]. Thus, clinical training is recommended to increase and maintain competencies among nurses [2]. Clinical training has been recognized as an essential part of continuing professional development for nursing professionals. Certain forms of clinical training are implemented often as the mandatory programs for obtaining and renewing licenses or other types of professional qualifications for registered nurses in a number of countries [3,4]. These qualifications also help ensure employment opportunities and job security among nursing professionals [5].

New graduate nurses are one of the major target groups of clinical training programs. Their specific training needs are focused primarily on bridging gaps between nursing theories and nursing practices since new graduate nurses often encounter professional dilemmas and psychological conflicts derived from discrepancies between theory-based education at nursing education institutions and clinical practice realities at health facilities [6–8]. Several earlier studies reported that clinical training programs for new graduate nurses contributed to increasing and diversifying their competencies (e.g. clinical skills, decision making skills, critical thinking, and leadership) [9–14]. It was also known that these impacts of clinical training programs are attributable to improved curricula, appropriate lengths of the clinical training periods, inter-departmental rotation systems, supportive cultures in workplaces [10,15,16], and capacities of preceptors [15–20]. A majority of these studies were literature reviews of small-scale quasi-experimental or descriptive studies. There are few large-scale experimental studies that identified causalities between new graduate nurses’ competencies and clinical training programs [9,13,14,21].

In Vietnam, completion of a nine-month clinical training is the requirement for a new graduate nurse to obtain a national nursing practicing certificate. This certificate is a professional qualification equivalent to a national nursing license in other countries. They are required to gain competencies to respond to increasingly diverse patients’ demands due to the country’s rapid epidemiologic transition and aging [22]. In addition, Vietnam is committed to producing an adequate number of competent nurses, to meet the Southeast Asian regional nursing professional needs under the Mutual Recognition Arrangement, the framework in which intra-regional mobility of nursing professionals is encouraged and promoted among the Association of South East Asian Nations (ASEAN) member states [23]. Yet, Vietnam is currently unable to meet even domestic nursing workforce needs and is suffering a critical shortage. The number of physicians, nurses, and midwives per 100,000 population in Vietnam (= 2.27) [24] remains smaller than its international threshold (= 4.45) [25], thus making the shortage of the domestic nursing workforce more significant than in other countries. In addition, parallel implementations of three different nursing pre-service education programs (i.e. a two-year vocational program, three-year diploma program, and four-year bachelor program) and the absence of nationally standardized curricula for pre-service education programs are likely to further prevent the quality of nursing professionals in the country from being adequately ensured.

In view of this critical situation, the Government of Vietnam requires new graduates of all three types of nursing educational institutions to complete nine-month clinical training to obtain a national nursing practicing certificate, by the Law on the Medical Examination and Treatment, a Vietnamese legal act that regulates health professional licensing [26]. In response to the government’s policy, the Basic Competency Standard for Vietnamese Nurses (the Competency Standard) was developed in view of the ASEAN competency standard by the Vietnamese Ministry of Health (MoH) [27]. However, the contents and quality of the clinical training programs are not necessarily in line with the basic competency standard but rather significantly differ according to recipient health facilities to which new-graduate nurses are assigned. This is attributable primarily to the absence of a nationally standardized curriculum for new graduate nurses’ clinical training.

To address the long-standing issues related to the quality assurance of the clinical training for new graduate nurses, the MoH, in collaboration with the Japan International Cooperation Agency (JICA), implemented the Project for Strengthening Clinical Training System for New-Graduate Nurses (the Project), during the period from 2016 to 2020 [28]. The Project developed and piloted a series of technical tools based on the Competency Standard [27]: specifically, (i) a standard curriculum and textbooks; (ii) a preceptor training manual; and (iii) clinical training management guidelines. The standard clinical training program for new graduate nurses (incl. a standard curriculum) was designed and further piloted at 20 provincial and city hospitals located in four provinces and one city in Vietnam [28]. Thus, the effectiveness of the standard clinical training program needs to be assessed to make an informed policy decision on the nationwide scaling-up of the post-graduate clinical training program. Furthermore, there is a need for a scientific contribution to estimate the effectiveness of standard clinical training programs in competencies required for new graduate nurses, beyond the Vietnamese context [9,13,14,21].

This study aimed to estimate the effectiveness of the standard clinical training program by employing a difference-in-differences (DID) approach to compare new graduate nurses’ competencies in the intervention group with those in the control group between pre- and post-clinical training stages. This study serves as the first study of its kind in Vietnam and one of the few studies worldwide that estimated the effectiveness of clinical training programs exclusively for new graduate nurses.

## Methods

### Study design and groups

DID, a study design characteristic of quasi-experimental longitudinal studies, is often used for estimating an impact of a public health intervention because employing a randomized controlled trial is neither feasible nor ethical [29]. In this study, DID was employed because the intervention involved the process of issuing a national nursing qualification [26].

In this study, a new graduate nurse is defined as an individual who graduated in the last 12 months from any of the three types of nursing education institutions and was willing to obtain a national nursing practicing certificate or had graduated earlier but became willing to obtain a national nursing practicing certificate.

The standard clinical training program was implemented at 9 hospitals in four provinces (Binh Dinh, Dien Bien, Dong Nai, and Vinh Phuc) and in one city (Hanoi) during the study period from April 2019 to May 2020. Therefore, all the nine hospitals were selected as the intervention group for this study.

Nghe An and Ho Chi Minh were purposively determined as the province and city from which the control group hospitals were sought. Of all the government hospitals in Nghe An and Ho Chi Minh, hospitals whose profiles were matched with those in the intervention group were identified and listed. Then, all the seven hospitals having new graduate nurses were selected from the list as the control group.

**Fig 1:**
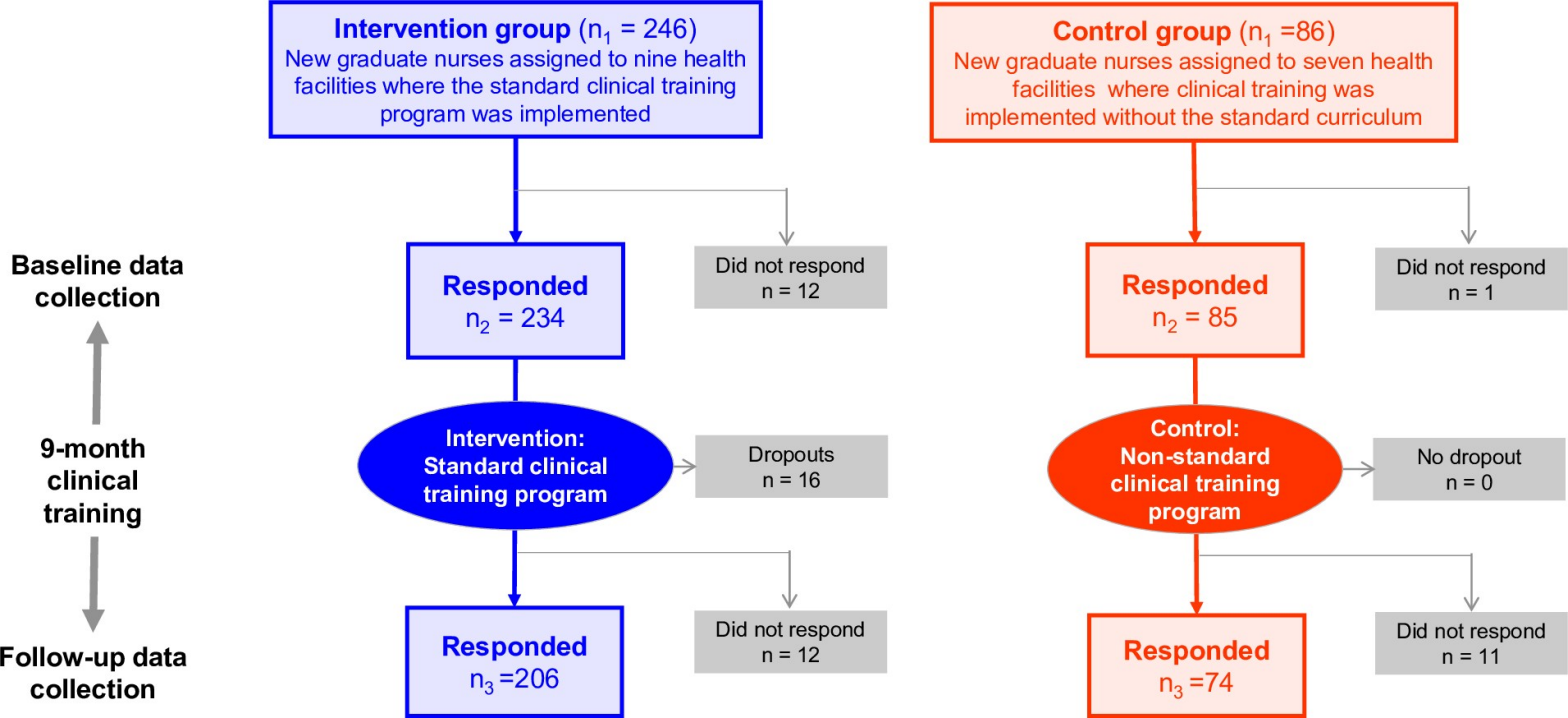
Flow diagram showing the number of study participants in the intervention and control groups.

Of the nine governmental hospitals in the intervention group, eight were provincial hospitals and one was a district hospital. Of the seven government hospitals in the control group, five and two were provincial hospitals and district hospitals, respectively. A total of 246 and 86 new graduate nurses (N = 332) at the nine hospitals in the intervention group and the seven hospitals in the control group were targeted, respectively (Fig 1).

### Intervention

The clinical training program developed by the Project was composed of three elements: (i) clinical training for new graduate nurses with a standardized curriculum based on the Competency Standard; (ii) support provided by trained receptors; and (iii) systematic management in accordance with the guidelines [30]. To facilitate the training of the intervention group, medical equipment (e.g., vein injection exercise kit) was provided after completing the intervention.

The duration of clinical training was pre-determined by the Law on the Medical Examination and Treatment as nine months. The standard clinical training curriculum set five goals to be achieved in line with the Competency Standard [27] which is composed of three domains. The three domains are further composed of 25 competencies with 110 competency indicators for measuring the levels of those competencies [27] (S1 File). The five types of capacities a new graduate nurse is required to achieve during clinical training are: (i) demonstrating evidence-based fundamental nursing skills; (ii) providing nursing care based on the principles of patient safety and infection control; (iii) demonstrating effective and proper communication, team working, and health education to patients and families; (iv) promoting quality improvement and effective resource management; and (v) providing nursing care in line with the related regulations and professional ethics. The clinical training curriculum also requires each new graduate nurse to complete a total of 1520 units during the nine-month period. Of the 1520 units, 76 and 1324 are designated as On-the-Job-Training (OJT) and Off-the-Job-Training (Off-JT), respectively. The rest (i.e. 120 units) are designated as self-learning sessions, such as preparation and review for both OJT and Off-JT. The curriculum of the clinical training program defines Off-JT as classroom-based lectures, discussions, group works, experiments, and practices, and OJT as ward-based practices involving patients (Tables 1 and 2).

**Table 1:** Composition of curriculum for clinical training for new graduate nurses.

Methods	Contents	Unit
**Theory and practice**	Orientation and nursing regulations	10
	Patient safety	16
	Basic nursing technique and patient care	12
	Patient care management	6
	First aid and emergency	12
	Communication, Consultation and teamwork	20
	**Sub-total**	**76**
**Practice at clinical departments**	Practicing at different clinical departments. Preceptors support new graduate nurses based on each nurse’s achievement level. Training facility can apply a rotation program according to a suitable schedule.	1,324
**Review, self-study, test, and evaluation**	Review	40
	Nursing process including care plan development	40
	Examination and evaluation	32
	Summary and closing	8
	**Sub-total**	**120**
**Total**		1,520

**Table 2:** Structure and contents of textbook to be used for OJT and Off-JT.

	Chapter		Section
**1**	**Orientation and nursing regulations**	1.1	Introduction of organization, regulations of the hospital Implementing clinical training, training program, clinical learning method for new nurses
		1.2	Basic competency standards for nurses in Vietnam
		1.3	Applying professional ethics for nurses in Vietnam to the practice of patient care
		1.4	Regulations on nursing and patient care
**2**	**Patient safety**	2.1	Application of standard precautions in patient care practice
		2.2	Prevention of medical adverse events errors
**3**	**Basic nursing technique and patient care**	3.1	Pain relief care
		3.2	Application of nursing process in patient care
		3.3	Receiving, transferring and discharging patients
		3.4	Vital sign monitoring
		3.5	Specimen collection for testing (blood, sputum, stool, urine)
		3.6	Hygiene care of patient
		3.7	Patient movement support
		3.8	Feeding support to patients
		3.9	Medication practice to patients
		3.10	Fluid infusion–blood transfusion techniques
		3.11	Monitoring volume of in and out fluid
		3.12	Wound and drainage tube care techniques
		3.13	Pressure ulcer prevention and care for patients
		3.14	Excretion care
**4**	**Patient care management**	4.1	Regulations on recording, managing medical records and care templates
		4.2	Medical equipment usage and management: monitor, infusion machine, injection pump, electrocardiograph
		4.3	Management of medicines and medical consumable supplies
**5**	**First aid, emergency**	5.1	Evaluation of comatose patient based on Glasgow Coma Scale
		5.2	Respiratory support and airway management techniques
		5.3	Basic emergency care for cardiac arrest
		5.4	Prevention and management of anaphylaxis
**6**	**Communication, consultation and teamwork**	6.1	Communication skills in patient care
		6.2	Health education
		6.3	Teamwork skills in health care

A preceptor training program was developed for experienced nurses who will be responsible for: (i) planning and providing for new graduate nurses with Off-JT; (ii) guiding and supporting them during OJT; and (iii) applying active learning methods, evidence-based practices, and proper assessments and evaluation for both Off-JT and OJT. The preceptor training program was designed to be a five-day course composed of 40 units and conducted by a qualified master trainer with a national certificate of clinical teaching methods.

Both the clinical training program for new graduate nurses and preceptor training program for experienced nurses were organized by a nurse manager at each hospital. When organizing and planning for the programs, the guidelines for the management of clinical training for new graduate nurses needed to be referred to and abided by. The guidelines provided nurse managers with practical guidance on how to plan for, implement, and evaluate both training programs. The guidelines recommend that OJT be implemented not exclusively at one department but at several major departments on a rotational basis (e.g. outpatient department, pediatric department, obstetric and gynecological department, and surgical department) to enable new graduate nurses to gain diverse practical experiences and skills.

### Outcomes

**Fig 2:**
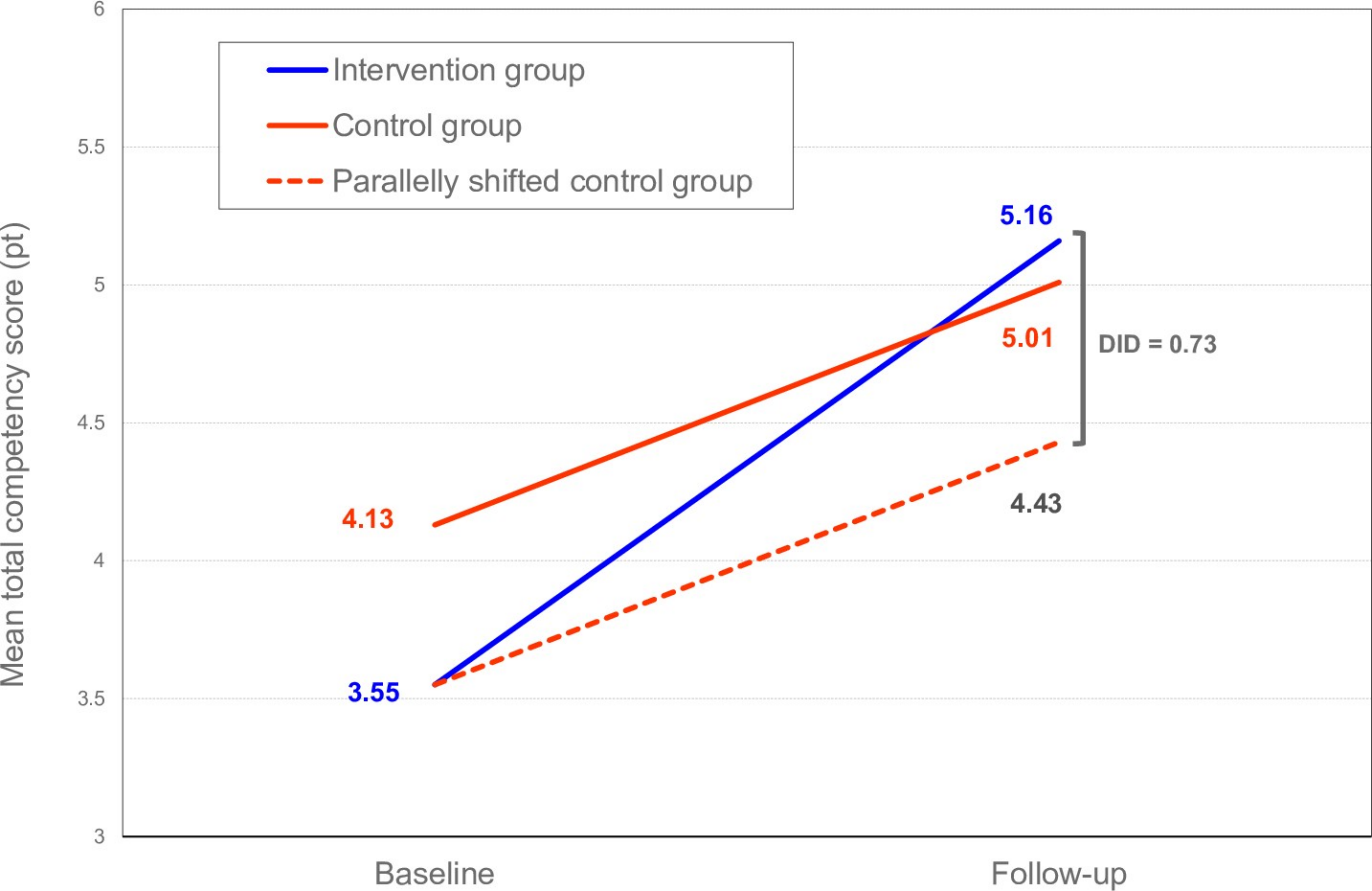
Difference-in-differences in mean increases in total competency scores.

The change in new graduate nurses’ competencies between the baseline (pre-clinical-training stage) and follow-up (post-clinical-training stage) was set as the outcome of the intervention. While there are diverse self-reporting competency assessment tools, none were readily available for nurses in Vietnam at the time of this study [31]. To assess the competency of new graduate nurses in Vietnam, a questionnaire based on the Competency Standard was developed (S2 and S3 Files). To assess the levels of 25 competencies, the scores for the 110 competency indicators were self-determined by new graduate nurses. The self-determined score for each indicator was marked based on a three-point Likert scale, i.e. “0 = *cannot do*,” “1 = *can do with assistance*,” and “2 = *can do independently*.” As shown in Fig 2, the number of the competency indicators differs from one competency to another. For instance, while the number of the competency indicators for “*Competency 1*: *demonstrate knowledge based on health and illness status of individuals*, *groups*, *and communities*” is two (the smallest), for “*Competency 4*: *utilize nursing process as framework for nursing plan and interventions*” the number of indicators is nine (the greatest). Therefore, a mean competency score of the composing indicators was calculated as the score for each competency (i.e. minimum 0, maximum 2 points). The final overall scores were calculated for respective new graduate nurses by summing up the scores of the 25 competencies (i.e. minimum 0, maximum 6 points, theoretically).

The competency standard was originally developed in Vietnamese and English in collaboration with Vietnamese and Australian nursing educators. This study team followed the original expression to develop a questionnaire. Other questions and scales were developed in English first, then translated into Vietnamese. Back translation was completed by a Vietnamese professional who was not engaged in the questionnaire development process.

Prior to the study, a pre-test was conducted for new graduate nurses in the hospitals that were not involved in both intervention and control groups to correct the expressions of questionnaire that were difficult to understand. To ensure the content validity of this questionnaire, the 110 competency indicators were assessed and confirmed through discussing and building a consensus among national and international nursing educators. An internal consistency analysis was performed to evaluate the reliability of this scale. The total scale’s internal consistency reported by Cronbach’s α was 0.98, and Cronbach’s α coefficients of the subscales corresponding to the three domains of the competency standard were 0.97 for domain1, 0.96 for domain2, and 0.87 for domain3.

### Data collection

Self-determined scores were collected using a web-based self-administered questionnaire. Prior to self-administration, study team members provided target new graduate nurses with guidance for accessing the web-based questionnaire and responding to questions. Unique IDs were developed for target nurses and corresponding QR codes were scanned using their personal mobile phones. Target nurses could then access the first page of the questionnaire regarding informed consent; only those who consented to their cooperation in this study could proceed to the next page and answer the questions. The starting dates of the clinical training program differed between hospitals due to their staff training calendars. For this reason, the baseline data were collected during a relatively long period, from April to September 2019, and follow-up data were collected during the period from December 2019 to May 2020, accordingly. Those who participated in the study at a slightly later stage were requested to retrospectively self-administer the questionnaire for baseline data by recalling their pre-clinical-training situations.

To identify the factors associated with increases in nurses’ competencies, two types of data in view of earlier studies were collected [10,15,16]. First, individual-specific data (e.g. age, sex, ethnicity, marital status, and type of pre-service education program) were collected from new graduate nurses in both groups. Second, clinical-training-facility-specific data (e.g. type of health facilities, number of nurses, number of beds, and employment statuses) were collected from hospital information and documentation systems from both groups at the post-clinical-training stage.

### Data analysis

To calculate DID in mean total competency score, first, intra-group differences in mean and standard deviation were compared between baseline and follow-up data. Then, the mean increases and standard deviations of competency scores were compared between the intervention and control groups.

In addition, to assess the magnitude and precision of the effect of the standard clinical training program, Cohen’s d as the effect size and 95% confidence intervals (95% CI) were calculated [32,33]. The effect sizes were interpreted with five categories, that is, <0 as an adverse effect, 0–0.20 as no effect, 0.20–0.50 as a small effect, 0.50–0.80 as an intermediate effect, and ≥0.80 as a large effect [32].

Lastly, multivariate analysis, namely the generalized linear model, were performed using the mean increase in competency score as the dependent variable because the characteristics of the two groups were different. The independent variables for multivariate analyses were selected based on the results of earlier studies, namely age, baseline score, type of pre-service education program, and contractual status during clinical training as the individual-specific independent variables, and type of health facility as the facility-specific independent variable [10,15,16]. All statistical analyses were performed using SPSS for Windows version 25 (IBM/SPSS Inc., Chicago, USA), and the effect size and 95% confidence intervals were calculated using Psychometrica [33].

### Ethical considerations

The study protocol was submitted, along with other necessary documents, such as questionnaires, to the Institutional Review Board (IRB) of Hanoi University of Public Health (HUPH), for its ethical clearance. The protocol was then approved accordingly (274/2019/YTCC-HD3). The study was conducted in accordance with the principles set out in the Helsinki Declaration and other regulatory bodies. Prior to distributing the web questionnaire code, the participants were verbally and adequately briefed on the study’s objectives, data collection procedure, and voluntary participation. In addition, all new graduate nurses who participated in the study were requested to read an informed consent form that was displayed on the first page of the web-based questionnaire. Only the target nurses who agreed to cooperate with this study by pressing the “approve” button were directed to the following question page. Even though target nurses approved their cooperation to this study, they could stop at any time before submitting the questionnaire. Target nurses were informed prior to the study that they could not withdraw their participation once submitting. The seven health facilities in the control group would receive the standard clinical training program after completion of this study.

## Results

### Characteristics of targets

Of the 332 targeted new graduate nurses, 280 participated in both the baseline and follow-up data collection (overall response rate of 84.3%). The 52 non-respondents included 16 dropout cases from the intervention group (Fig 1). As shown in Table 3, ethnic group, type of pre-service education program, contractual status with clinical training facility, and type of health facility were significantly different between the two groups.

**Table 3:** Characteristics of study participants.

		Intervention group (n = 206)		Control group (n = 74)		*P-*value
**Individual-specific variables**						
Age, years	Mean (SD)	23.4	(2.73)	23.5	(2.98)	0.703^a^
Gender	Male	35	17.0%	10	13.5%	0.485^b^
	Female	171	83.0%	64	86.5%	
Ethnicity	Kinh	187	90.8%	73	98.6%	0.024^b^
	Others	19	9.2%	1	1.4%	
Type of pre-service education program	Intermediate	10	4.9%	4	5.4%	< 0.001^b^
	College	171	83.4%	41	55.4%	
	University or higher	24	11.7%	29	39.2%	
Contractual status with clinical training facility	Permanent contract	71	34.5%	16	21.6%	0.044^b^
	Fix-term contract	94	45.6%	46	62.2%	
	Internship	41	19.9%	12	16.2%	
**Clinical-training-facility-specific variables**						
Type of health facility	Provincial hospital	200	97.1%	61	82.4%	< 0.001^b^
	District hospital	6	2.9%	13	17.6%	
Number of beds	0–999	39	18.9%	19	25.7%	0.466^b^
	1000–1499	99	48.1%	32	43.2%	
	1500–1999	68	33.0%	23	31.1%	
Number of nurses	0–499	133	64.6%	51	68.9%	0.498^b^
	500–999	73	35.4%	23	31.1%	

^a^ Mann-Whitney U test.

^b^ Chi-square test.

### Difference-in-differences in mean total competency score

As shown in Table 4, mean total competency score in the intervention group significantly increased from 3.55 (SD 1.22) at baseline to 5.16 (SD 0.91) at follow-up, with a large effect size (Cohen’s d = 1.50, 95% CI 1.19 to 1.81).

**Table 4:** Comparison of mean, standard deviation, and effect size of total competency scores between intervention and control groups.

	Intervention group (n = 206)					Control group (n = 74)					Mean increase in competency scores between intervention-control groups					Effect size ^b^
	Baseline		Follow up		Cohen’s d (95% CI) ^a^	Baseline		Follow up		Cohen’s d (95% CI) ^a^	Intervention		Control		Cohen’s d (95% CI) ^a^	
	Mean	(SD)	Mean	(SD)		Mean	(SD)	Mean	(SD)		Mean increase	(SD)	Mean increase	(SD)		
**Competency Score** [min 0- max 2 pt]																
1 Data collection	1.03	(0.62)	1.59	(0.50)	0.99 (0.71–1.28)	1.20	(0.53)	1.64	(0.45)	0.90 (0.42–1.37)	0.57	(0.77)	0.44	(0.60)	0.17 (-0.09–0.44)	No
2 Assessment	0.88	(0.49)	1.55	(0.47)	1.40 (1.09–1.70)	1.12	(0.46)	1.52	(0.41)	0.92 (0.44–1.40)	0.67	(0.63)	0.40	(0.52)	0.45 (0.18–0.72)	Small
3 Nursing diagnosis	1.02	(0.51)	1.65	(0.48)	1.27 (0.97–1.57)	1.23	(0.47)	1.60	(0.45)	0.80 (0.33–1.28)	0.63	(0.63)	0.37	(0.54)	0.43 (0.16–0.70)	Small
4 Nursing plan development	1.13	(0.47)	1.71	(0.34)	1.41 (1.11–1.72)	1.29	(0.37)	1.65	(0.35)	1.00 (0.52–1.48)	0.59	(0.52)	0.36	(0.46)	0.44 (0.17–0.71)	Small
5 Privacy protection	1.45	(0.49)	1.85	(0.29)	0.99 (0.70–1.28)	1.55	(0.40)	1.84	(0.29)	0.83 (0.36–1.31)	0.40	(0.55)	0.30	(0.45)	0.19 (-0.07–0.46)	No
6 Proper care delivery	1.37	(0.50)	1.89	(0.23)	1.34 (1.03–1.64)	1.38	(0.42)	1.82	(0.33)	1.17 (0.67–1.66)	0.53	(0.53)	0.44	(0.46)	0.18 (-0.09–0.44)	No
7 Medication administration	1.32	(0.45)	1.81	(0.23)	1.37 (1.07–1.68)	1.43	(0.39)	1.74	(0.30)	0.89 (0.41–1.37)	0.49	(0.47)	0.31	(0.42)	0.39 (0.12–0.66)	Small
8 Care continuity	1.18	(0.55)	1.74	(0.39)	1.18 (0.88–1.47)	1.34	(0.41)	1.69	(0.37)	0.90 (0.42–1.37)	0.56	(0.59)	0.35	(0.53)	0.37 (0.11–0.64)	Small
9 First aids	1.01	(0.50)	1.65	(0.41)	1.40 (1.10–1.70)	1.20	(0.44)	1.57	(0.41)	0.87 (0.39–1.35)	0.64	(0.55)	0.37	(0.52)	0.49 (0.22–0.76)	Small
10 Rapport with patients	1.43	(0.58)	1.88	(0.26)	1.00 (0.71–1.29)	1.70	(0.40)	1.81	(0.38)	0.28 (-0.18–0.74)	0.45	(0.65)	0.11	(0.53)	0.54 (0.27–0.81)	**Intermediate**
11 Interpersonal communication	1.32	(0.55)	1.77	(0.36)	0.97 (0.68–1.26)	1.56	(0.49)	1.79	(0.35)	0.54 (0.08–1.00)	0.45	(0.63)	0.22	(0.53)	0.38 (0.11–0.65)	Small
12 ICT Skills	1.21	(0.61)	1.75	(0.43)	1.02 (0.73–1.31)	1.47	(0.53)	1.79	(0.37)	0.70 (0.23–1.17)	0.54	(0.71)	0.32	(0.59)	0.33 (0.06–0.59)	Small
13 Intelligible explanation	1.11	(0.56)	1.71	(0.44)	1.19 (0.90–1.49)	1.26	(0.54)	1.64	(0.47)	0.75 (0.28–1.22)	0.60	(0.64)	0.39	(0.64)	0.34 (0.07–0.60)	Small
14 Health education	0.88	(0.50)	1.53	(0.50)	1.30 (1.00–1.60)	1.06	(0.48)	1.50	(0.44)	0.96 (0.48–1.44)	0.65	(0.61)	0.44	(0.58)	0.35 (0.08–0.61)	Small
15 Teamworking	1.44	(0.41)	1.83	(0.26)	1.14 (0.84–1.43)	1.58	(0.32)	1.79	(0.30)	0.68 (0.21–1.15)	0.39	(0.50)	0.21	(0.38)	0.37 (0.10–0.63)	Small
16 Documentation	1.20	(0.50)	1.74	(0.35)	1.25 (0.95–1.55)	1.34	(0.38)	1.69	(0.34)	0.97 (0.49–1.45)	0.54	(0.54)	0.35	(0.43)	0.38 (0.11–0.64)	Small
17 Care management	1.03	(0.43)	1.60	(0.41)	1.36 (1.05–1.66)	1.24	(0.43)	1.56	(0.41)	0.76 (0.29–1.23)	0.57	(0.56)	0.33	(0.49)	0.44 (0.17–0.71)	Small
18 Medical equipment management	0.93	(0.49)	1.52	(0.52)	1.17 (0.87–1.46)	0.98	(0.47)	1.49	(0.50)	1.05 (0.57–1.54)	0.59	(0.67)	0.51	(0.59)	0.12 (-0.15–0.39)	No
19 Resource management	0.80	(0.58)	1.45	(0.63)	1.07 (0.78–1.37)	1.01	(0.52)	1.45	(0.59)	0.79 (0.32–1.26)	0.65	(0.79)	0.44	(0.71)	0.27 (0.01–0.54)	Small
20 Working environment	1.29	(0.49)	1.81	(0.29)	1.29 (0.99–1.59)	1.44	(0.42)	1.73	(0.36)	0.74 (0.27–1.21)	0.51	(0.53)	0.29	(0.48)	0.44 (0.17–0.70)	Small
21 Quality improvement	1.02	(0.49)	1.60	(0.44)	1.25 (0.95–1.54)	1.24	(0.40)	1.58	(0.45)	0.80 (0.33–1.27)	0.58	(0.61)	0.35	(0.55)	0.39 (0.13–0.66)	Small
22 Evidence based practice	0.63	(0.54)	1.26	(0.64)	1.06 (0.77–1.36)	0.85	(0.52)	1.26	(0.55)	0.77 (0.29–1.24)	0.63	(0.74)	0.41	(0.66)	0.31 (0.05–0.58)	Small
23 Professional development	1.21	(0.50)	1.77	(0.34)	1.31 (1.01–1.61)	1.49	(0.39)	1.68	(0.37)	0.50 (0.04–0.96)	0.57	(0.58)	0.19	(0.52)	0.66 (0.39–0.94)	**Intermediate**
24 Legal compliance	1.44	(0.52)	1.89	(0.24)	1.11 (0.82–1.41)	1.65	(0.37)	1.78	(0.33)	0.37 (-0.09–0.83)	0.45	(0.56)	0.14	(0.46)	0.58 (0.31–0.85)	**Intermediate**
25 Moral responsibility	1.27	(0.60)	1.78	(0.41)	0.99 (0.70–1.28)	1.49	(0.47)	1.74	(0.37)	0.59 (0.13–1.06)	0.51	(0.71)	0.25	(0.61)	0.39 (0.12–0.66)	Small
**Total Competency Score** [min 0- max 6 pt]	3.55	(1.22)	5.16	(0.91)	1.50 (1.19–1.81)	4.13	(0.93)	5.01	(0.91)	0.96 (0.48–1.44)	1.61	(1.42)	0.88	(1.17)	0.53 (0.26–0.80)	**Intermediate**

^a^ Effect size and 95% confidence interval were calculated by using Psychometrica (33).

^b^ Effect size was categorized into five levels: Adverse Effect: <0; No Effect: 0─0.2; Small Effect: 0.2─0.5; Intermediate Effect: 0.5─0.8, and Large Effect: ≥ 0.8.

Mean increase in total competency scores of the intervention group (1.61, SD 1.42) was greater than that of the control group (0.88, SD 1.17). Cohen’s d was 0.53 (95% CI 0.26 to 0.80), indicating an intermediate effect (Table 4).

Fig 2 presents DID of mean total competency scores. The estimated DID was 0.73 (= 5.16–4.43) (Fig 2).

**Fig 3:**
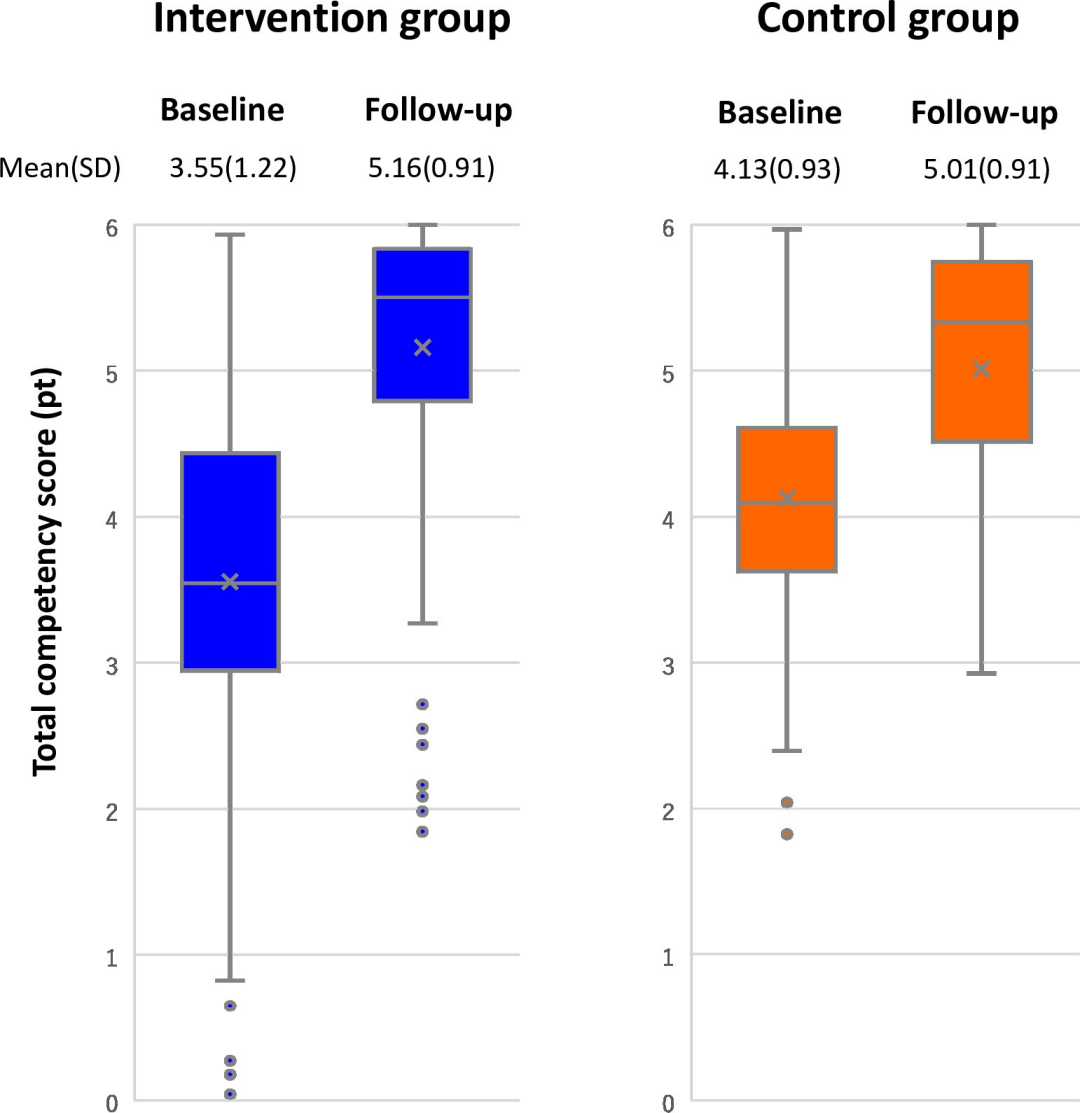
Comparison of mean and standard deviation of total competency scores between groups.

Reduction in standard deviation in the intervention group was greater than in the control group (Table 4 and Fig 3).

### Comparison of mean of individual competency scores between groups

A greater increase in competency scores of the intervention group than that of the control group was detected systematically in all 25 competencies (Table 4). In three of the 25 competencies, an intermediate effect (0.2 < Cohen’s d <0.5) was detected. Cohen’s d for “professional development,” “legal compliance,” and “rapport with patients” were 0.66 (95% CI 0.39 to 0.94), 0.58 (95% CI 0.31 to 0.85), and 0.54 (95% CI 0.27 to 0.81), respectively. Cohen’s d for “medical equipment management,” “data collection,” and “proper care delivery” were 0.12 (95% CI -0.15 to 0.39), 0.17 (95% CI -0.09 to 0.44), and 0.18 (95% CI -0.09 to 0.44), respectively, indicating no effect.

### Determinants of mean increase in competency scores

Table 5 shows the results of multivariate analyses. They indicated that implementation of the standard clinical training produced a slightly positive association with a mean increase in total competency score (β = 0.04), yet without significance (*P* = 0.321). However, type of health facility produced a significantly positive association (β = 0.11, *P* = 0.007), and baseline competency score had a significantly negative association with a mean increase in competency scores (β = -0.75, *P* < 0.001).

**Table 5:** Determinants of the mean increase in total competency scores.

Dependent variable	*β*	*P*-value
Standardized clinical training (intervention)	0.04	0.321
Age	-0.05	0.199
Baseline score	-0.75	<0.001
Pre-service training ^a^	0.00	0.940
Contractual status ^b^	0.06	0.118
Type of health facility ^c^	0.11	0.007

Generalized Linear Model was performed as multivariate analysis: Adjusted R^2^ = 0.58, F = 65.44.

^a^ 1 = intermediate, 2 = college, 3 = university or upper.

^b^ 1 = intern, 2 = fix-term contract, 3 = permanent.

^c^ Dichotomous variable, where provincial hospital = 1 and district hospital = 0.

## Discussion

### Effectiveness of standard clinical training program

DID was performed to estimate the effectiveness of the clinical training system for new graduate nurses in Vietnam. The results of DID indicate that the increase in the mean total competency score of new graduate nurses was significantly greater in the intervention group than in the control group with an intermediate effect size (Fig 2 and Table 4). However, multivariable analysis showed that clinical training has a positive association with the mean increase in total competency score but without significance.

These results imply that the novel standard clinical training program is likely to be effective, therefore confirming several earlier studies [9–14]. As the earlier studies employed cross-sectional descriptive designs with small sample sizes [9,13,14,21], this study employed a quasi-experimental longitudinal design with an adequate sample size, therefore providing more reliable effectiveness.

A greater increase in the mean total competency score of new graduate nurses in the intervention group could be caused by specific components of the intervention. In the intervention group, nurse managers of the health facilities planned for and implemented both standard clinical training programs and preceptor training programs. When planning for standard clinical training programs, they particularly attempted to ensure three key operational elements: (i) use of competency-based curriculum; (ii) readily available preceptors; and (iii) a set of interventions on new graduate nurses (i.e. orientation, OJT, Off-JT, and evaluation). These operational components are likely to have synchronously contributed to achieving the greater increase in mean total competency scores in the intervention group. These types of clinical training components were identified as the factors that increase competencies among new graduate nurses in earlier studies [10,15,16]. In terms of training duration, six months was reported as an appropriate duration for nurses to obtain clinical leadership competencies [10]. However, the results of our study suggest that nine months, which the MoH set as the standard clinical training duration in line with the Law on the Medical Examination and Treatment, is likely to be the appropriate clinical training duration for new graduate nurses to obtain basic clinical competencies.

In three of the 25 competencies (“professional development,” “legal compliance,” and “rapport with patients”), intermediate effects of the standard clinical training were detected, while no effect was detected in another three competencies (“medical equipment management,” “data collection,” and “proper care delivery”). All the three competencies in which intermediate effects were detected are related to personal and professional values that are generally difficult to change [34,35] and could change when systematic education is involved [36,37]. Thus, the standard clinical training program might have successfully enabled new graduate nurses to increase their competencies related to personal and profession values by providing valuable opportunities to cultivate and further change new graduate nurses’ mindsets on nursing professionalism. On the other hand, competencies related to technical knowledge and skills could increase even in the control group through traditional clinical training programs that are neither standardized nor systematic, implying that technical knowledge and skills could be obtained regardless of the type and mode of clinical training received. However, self-reported competency assessments tend to result in greater increases in competencies related to technical knowledge and skill [38]; thus, it might be necessary to interpret this as one of the limitations of self-reported competencies assessments.

Furthermore, the reduction in standard deviation of competencies scores in the intervention group was greater than that in the control group (Fig 3). This implies that the standard clinical training produced not only more highly competent nursing professionals but also more homogeneously competent nursing professionals. There are very few studies that have addressed and reported the production of homogeneously competent nursing professionals through clinical training. Particularly in countries where multiple nursing pre-service education programs are ongoing in parallel (incl. Vietnam), homogenization or standardization of the nursing workforce has been a key challenge, as the levels of competencies among new graduate nurses largely differ according to the type of pre-service education programs they completed. The reduction in standard deviation of competency scores shows the effectiveness of standard clinical training.

The results of multivariate analyses indicate that intervening (i.e. with novel standard clinical training or traditional clinical training) was not significantly associated with the increase in total competency scores. On the other hand, type of health facility, specifically having clinical training at a provincial hospital, was positively associated with a greater mean increase in total competency score. This implies that provincial hospitals are more capable than district hospitals of undertaking clinical training for new graduate nurses because of their established clinical training systems and more available resources. Another possible interpretation of this is that some competencies, defined in the Competency Standard and measured in this study, might not be appropriate and applicable to new graduate nurses assigned to district hospitals. This is because the types and quality of knowledge, skills, and practices required differ by the level and function of health facilities [39,40]. In this context, it might be necessary to define facility-level-specific or facility-function-specific competencies to develop a more customized clinical training program, while recognizing the importance of homogenization and standardization of the nursing workforce, particularly when addressing new graduate nurses.

This study found that a mean increase in total competency scores was significantly associated, not with type of pre-service education program, but with baseline competency scores, even though several earlier studies reported significant associations between type of pre-service education programs and outcomes of patient care [41,42]. This implies that those with greater competency scores at the start of clinical training were likely to have limited room to increase their competencies. Capacities and base competencies of new graduate nurses are diverse for two reasons: first, as aforementioned, new graduate nurses have completed different types of pre-service education programs (i.e. two-year, three-year, or four-year programs). Second, the absence of nationally standardized curricula for respective types of pre-service education programs allows each education institution to develop its own original pre-service education curricula.

### Policy implications

The results of this study suggest that the introduction of a standard clinical training system could contribute to homogenizing and standardizing new graduate nurses’ competencies before they obtain national nursing practicing certificates. However, the objectives and roles of clinical training should not be limited to filling gaps in competencies between differently educated new graduate nurses but should go beyond it.

The Government of Vietnam is currently planning to introduce national examinations to be taken upon the completion of pre-service educational program to obtain a nursing practicing certificate [43]. Thus, respective education institutions will be intervened by adjusting their curricula to suit the national examination, or developing and applying national standard pre-service curricula. As it stands, the absence of standard curricula for pre-service education makes it difficult to enable new graduate nurses to smoothly move into the nursing workforce. It is crucial to build the seamless professional development pathway from pre-service education to clinical training and licensing to ensure a smoother transfer from nursing student to nursing professional.

### Limitations of the study

The results of multivariate analyses showed no significant association between intervention (standard clinical training or traditional clinical training) and increase in competency scores. Therefore, it is necessary to better design future studies to more precisely estimate the effectiveness of the clinical training program.

The Competency Standard was applied to the scale of self-reported assessment of new graduate nurses’ competencies, however, it had not been developed for this specific use. Thus, it is necessary to develop a nursing competency measurement tool to more precisely estimate new nurses’ competencies in Vietnam.

In addition, because the data collection period took about one year, nurses who trained in the health facility at control sites might have obtained information regarding this training program or this study. Influence of data contamination should be considered when interpreting this study’s results.

## Conclusion

The results of this study indicated that new graduate nurses who had participated in the standard clinical training program achieved a significantly greater increase in competency scores than those who had not. The increase in new graduate nurses’ competencies was attributed not only to clinical-training-facility factors (e.g. type of health facility) but also to new graduate nurses’ individual factors (e.g. their pre-clinical training competencies). It is recommended that the design of clinical training programs be adjusted and customized to the roles and functions of respective health facilities, at least to a certain extent. However, more importantly, the absence of standard curricula for pre-service education needs to be urgently addressed to maximize the effectiveness of clinical training for new graduate nurses. A further study is needed to more precisely examine the attribution of standard clinical training to an increase in new graduate nurses’ competencies.

## Supporting information

S1 File Basic competency standard for Vietnamese nurses. (PDF)

S2 File Baseline questionnaire in English and Vietnamese. (PDF)

S3 File Follow-up questionnaire in English and Vietnamese. (PDF)

## References

[1] World Health Organization. World health statistics overview 2019: Monitoring health for the SDGs, sustainable development goals. 2019 [cited 2020 December 13]. Available from: https://apps.who.int/iris/handle/10665/311696.

[2] Joint Commission on Accreditation of Healthcare Organization. Health care at the Crossroads. 2002 [cited 2020 December 13]. Available from: https://www.jointcommission.org/-/media/deprecated-unorganized/imported-assets/tjc/system-folders/topics-.

[3] The ASEAN Joint Coordinating Committee on Nursing. Requirements for continuous professional development. 2012 [cited on 2020 December 13]. Available from: https://asean.org/wp-content/uploads/2012/05/02-Requirements-for-CPD-AJCCN-25.pdf.

[4] Efendi F, Nursalam N, Kurniati A, Gunawan J. Nursing qualification and workforce for the Association of Southeast Asian Nations Economic Community. Nurs Forum. 2018;53(2):197–203. doi: 10.1111/nuf.12243 29359337

[5] Sonoda M, Syhavong B, Vongsamphanh C, Phoutsavath P, Inthapanith P, Rotem A, et al. The evolution of the national licensing system of health care professionals: A qualitative descriptive case study in Lao People’s Democratic Republic. Hum Resour Health. 2017;15(1):51. doi: 10.1186/s12960-017-0215-2 28784154 PMC5547512

[6] Kramer M. Why does reality shock continue? In: McCloskey JC, Grace HK, editors. Current issues in nursing. Malden: Blackwell Scientific; 1983. pp. 891–903.

[7] Wakefield E. Is your graduate nurse suffering from transition shock? J Periop Nurs. 2018;31(1):47–50.

[8] Duchscher JE. Transition shock: The initial stage of role adaptation for newly graduated registered nurses. J Adv Nurs. 2009;65(5):1103–13. doi: 10.1111/j.1365-2648.2008.04898.x 19183235

[9] Edwards D, Hawker C, Carrier J, Rees C. A systematic review of the effectiveness of strategies and interventions to improve the transition from student to newly qualified nurse. Int J Nurs Stud. 2015;52(7):1254–68. doi: 10.1016/j.ijnurstu.2015.03.007 26001854

[10] Chappell KB, Richards KC. New graduate nurses, new graduate nurse transition programs, and clinical leadership skill: A systematic review. J Nurses Prof Dev. 2015;31(3):128–37; quiz E8. doi: 10.1097/NND.0000000000000159 25993451

[11] Cheng CY, Tsai HM, Chang CH, Liou SR. New graduate nurses’ clinical competence, clinical stress, and intention to leave: A longitudinal study in Taiwan. Sci World J. 2014; e748389. 10.1155/2014/748389 PMC3926320 24672363

[12] AL-Dossary R, Kitsantas P, Maddox PJ. The impact of residency programs on new nurse graduates’ clinical decision-making and leadership skills: A systematic review. Nurse Educ Today. 2014;34(6):1024–8. doi: 10.1016/j.nedt.2013.10.006 24183633

[13] Anderson G, Hair C, Todero C. Nurse residency programs: An evidence-based review of theory, process, and outcomes. J Prof Nurs. 2012;28(4):203–12. doi: 10.1016/j.profnurs.2011.11.020 22818190

[14] Edwards D, Hawker C, Carrier J, Rees C. The effectiveness of strategies and interventions that aim to assist the transition from student to newly qualified nurse. JBI Libr Syst Rev. 2011;9(53):2215–323. doi: 10.11124/01938924-201109530-00001 27820299

[15] Rush KL, Janke R, Duchscher JE, Phillips R, Kaur S. Best practices of formal new graduate transition programs: An integrative review. Int J Nurs Stud. 2019;94:139–58. doi: 10.1016/j.ijnurstu.2019.02.010 30965203

[16] Innes T, Calleja P. Transition support for new graduate and novice nurses in critical care settings: An integrative review of the literature. Nurse Educ Pract. 2018;30:62–72. doi: 10.1016/j.nepr.2018.03.001 29571106

[17] Chen F, Liu Y, Wang X, Dong H. Transition shock, preceptor support and nursing competency among newly graduated registered nurses: A cross-sectional study. Nurse Educ Today. 2021;102:e104891. 10.1016/j.nedt.2021.104891 33866200

[18] Ke YT, Kuo CC, Hung CH. The effects of nursing preceptorship on new nurses’ competence, professional socialization, job satisfaction and retention: A systematic review. J Adv Nurs. 2017;73(10):2296–305. doi: 10.1111/jan.13317 28398636

[19] Whitehead B, Owen P, Holmes D, Beddingham E, Simmons M, Henshaw L, et al. Supporting newly qualified nurses in the UK: A systematic literature review. Nurse Educ Today. 2013;33(4):370–7. doi: 10.1016/j.nedt.2013.01.009 23416083

[20] Theisen JL, Sandau KE. Competency of new graduate nurses: A review of their weaknesses and strategies for success. J Contin Educ Nurs. 2013;44(9):406–14. doi: 10.3928/00220124-20130617-38 23799789

[21] Aldosari N, Pryjmachuk S, Cooke H. Newly qualified nurses’ transition from learning to doing: a scoping review. Int J Nurs Stud. 2021;113:e103792. doi: 10.1016/j.ijnurstu.2020.103792 33120135

[22] United Nations, Department of Economic and Social Affairs, Population Division. World population prospects 2019, Online Edition. Rev. 1. 2019 [cited 2020 December 13]. Available from: https://population.un.org/wpp/Download/Standard/Population/.

[23] ASEAN Secretariat. ASEAN economic community blueprint. 2008. [cited 2020 December 13]. Available from: https://asean.org/wp-content/uploads/archive/5187-10.pdf.

[24] World Health Organization and ICN. State of the world’s nursing report—2020. 978-92-4-000327-9. 2020 [Cited 2020 April 6] Available from: https://www.who.int/publications/i/item/9789240003279.

[25] World Health Organization. Global strategy on human resources for health: Workforce 2030. Geneva: WHO; 2016. [cited 2020 December 13] available from: https://apps.who.int/iris/bitstream/handle/10665/250368/9789241511131-eng.pdf?sequence=1.

[26] Ministry of Health Vietnam. Law on the medical examination and treatment. 40/2009/QH12. 2010 [cited 2020 December 13]. Available from: https://vss.gov.vn/english/legal/pages/default.aspx?ItemID=3551.

[27] Ministry of Health Vietnam. The basic competency standard for Vietnamese nurses. 1352/QĐ-BYT. 2012 [cited 2020 December 13]. Available from: http://asttmoh.vn/ban-hanh-chuan-nang-luc-co-ban-cua-dieu-duong-viet-nam/.

[28] Japan International Cooperation Agency. JICA project for strengthening clinical training system for new graduate nurses, Vietnam. 2016 [cited 2020 December 13]. Available from: https://www.jica.go.jp/project/vietnam/038/index.html.

[29] Wing C, Simon K, Bello-Gomez RA. Designing difference in difference studies: Best practices for public health policy research. Annu Rev Public Health. 2018;39:453–69. doi: 10.1146/annurev-publhealth-040617-013507 29328877

[30] Ministry of Health Vietnam. Clinical training system for new graduate nurses. 2019 [cited 2020 December 13]. Available from: http://asttmoh.vn/chiase/tai-lieu-dao-tao-thuc-hanh-lam-sang-cho-dieu-duong-moi/.

[31] Wilkinson CA. Competency assessment tools for registered nurses: An integrative review. J Contin Educ Nurs. 2013;44(1):31–7. doi: 10.3928/00220124-20121101-53 23413446

[32] Nakagawa S, Cuthill IC. Effect size, confidence interval and statistical significance: A practical guide for biologists. Biol Rev Camb Philos Soc. 2007;82(4):591–605. doi: 10.1111/j.1469-185X.2007.00027.x 17944619

[33] Lenhard W, Lenhard A. Calculation of effect sizes. 2016. [cited 2020 December 13]. Available from: https://www.psychometrica.de/effect_size.html. Germany. Dettelbach. Psychometrica. doi: 10.13140/RG.2.2.17823.92329

[34] Lombarts KM, Plochg T, Thompson CA, Arah OA. Measuring professionalism in medicine and nursing: Results of a European survey. PLOS ONE. 2014;9(5):e97069. doi: 10.1371/journal.pone.0097069 24849320 PMC4029578

[35] Parandeh A, Khaghanizade M, Mohammadi E, Mokhtari-Nouri J. Factors influencing development of professional values among nursing students and instructors: A systematic review. Glob J Health Sci. 2014;7(2):284–93. doi: 10.5539/gjhs.v7n2p284 25716397 PMC4796667

[36] O’Tuathaigh CMP, Nadhirah Idris A, Duggan E, Costa P, Costa MJ. Medical students’ empathy and attitudes towards professionalism: Relationship with personality, specialty preference and medical programme. PLOS ONE. 2019;14(5):e0215675. doi: 10.1371/journal.pone.0215675 31048851 PMC6497245

[37] Rassin M. Nurses’ Professional and Personal Values. Nurs Ethics. 2008;15:614–30. doi: 10.1177/0969733008092870 18687816

[38] Hengstberger-Sims C, Cowin LS, Eagar SC, Gregory L, Andrew S, Rolley J. Relating new graduate nurse competence to frequency of use. Collegian. 2008;15(2):69–76. doi: 10.1016/j.colegn.2008.02.003 18567478

[39] Brown RA, Crookes PA. What are the ’necessary’ skills for a newly graduating RN? Results of an Australian survey. BMC Nurs. 2016;15:23. doi: 10.1186/s12912-016-0144-8 27051351 PMC4820976

[40] Stanyon MR, Goldberg SE, Astle A, Griffiths A, Gordon AL. The competencies of Registered Nurses working in care homes: A modified Delphi study. Age Ageing. 2017;46(4):582–8. doi: 10.1093/ageing/afw244 28064168 PMC5859996

[41] Aiken LH, Sloane DM, Bruyneel L, Van den Heede K, Griffiths P, Busse R, et al. Nurse staffing and education and hospital mortality in nine European countries: A retrospective observational study. Lancet. 2014;383(9931):1824–30. doi: 10.1016/S0140-6736(13)62631-8 24581683 PMC4035380

[42] Audet LA, Bourgault P, Rochefort CM. Associations between nurse education and experience and the risk of mortality and adverse events in acute care hospitals: A systematic review of observational studies. Int J Nurs Stud. 2018;80:128–46. doi: 10.1016/j.ijnurstu.2018.01.007 29407346

[43] Ministry of Health Vietnam. Comment for draft of Law on the Medical Examination and Treatment. 2021. [cited 2021 April 23]. Available from: https://moh.gov.vn/gop-y-du-thao-van-ban?p_p_id=gydt_WAR_gydtportlet&p_p_lifecycle=0&p_p_state=normal&p_p_mode=view&p_p_col_id=row-0-column-2&p_p_col_count=1&_gydt_WAR_gydtportlet_jspPage=%2Fhtml%2Fgydt%2Fproposition_feedback%2Fview_proposition_details.jsp&_gydt_WAR_gydtportlet_redirect=%2Fgop-y-du-thao-van-ban&p_r_p_564233524_propositionId=682828.

