# Supplementary material for: Effectiveness of a standard clinical training program in new graduate nurses’ competencies in Vietnam: A quasi-experimental longitudinal study with a difference-in-differences design
Source: PLoS One. 2021 Jul 9;16(7):e0254238. doi: 10.1371/journal.pone.0254238 (PMC8270421; doi:10.1371/journal.pone.0254238)
Supplement: S1 File — (PDF) [file pone.0254238.s001.pdf]

Table1. Basic Competency Standard for Vietnamese Nurses [a]

Domain	Competency		Competency indicator
**Domain 1: Patient care**			
	1 Data collection	1	Identify the health need of the individuals, families, population groups and/or communities. (hereafter refers to patients/clients)
		2	Explain the health status of the clients/groups.
	2 Assessment	3	Collect and analyze information about health issues, disease to determine health problem and illness of individuals, families and communities.
		4	Make decisions of care for patients, families and communities safely and effectively.
		5	Perform nursing interventions to support individuals, families and communities to respond to the problem health/disease in accordance with cultural beliefs of the patient, patient family.
		6	Monitor the evolution of nursing interventions performed.
	3 Nursing diagnosis	7	Analyze and identify priority patients/clients' needs of care.
		8	Carry out appropriate nursing care to respond to priority needs/problems.
	4 Nursing plan development	9	Perform comprehensive and systematic nursing assessment.
		10	Collect and record patients'/clients' information appropriately.
		11	Analyze and explain the information exactly.
		12	Formulate a health needs based care plan on the basis of collaboration and agreement with patients/clients, their family and team other members on the priority issues and clients' expectations.
		13	Explain nursing interventions for patients, families and implement planned nursing care in a manner that ensures safety, comfort and efficiency for patients.
		14	Instruct patient/family to perform appropriate self-care.
		15	Evaluate and adjust care plan based on the patient's health condition and expected outcomes.
		16	Perform necessary support for discharged patients.
		17	Provide health education on disease prevention to patients.
	5 Privacy protection	18	Deploy safety measures in all aspects of patients/clients care.
		19	Create a convenient environment for patients.
		20	Ensure privacy while providing care to patients.
	6 Proper care delivery	21	Comply with steps of nursing process in the professional scopes.
		22	Implement nursing techniques skillfully.
		23	Follow the rules of infection control.
	7 Medication administration	24	Take a complete patient's drug history.
		25	Comply with rules while administering medication to patients.
		26	Explain and instruct patient about the drugs that is given to her/him.
		27	Detect and perform necessary intervention if the patient had any drug reactions and report promptly to doctor and nurse in charge.
		28	Be aware of potential drug - drug or drug-food interactions.
		29	Evaluate the effects of medication.
		30	Document and inform well drugs administered to patient.
	8 Care continuity	31	Hand over the patient's status to the next care team appropriately.
		32	Collaborate with patients, families and other team members effectively to ensure continuity of care.
		33	Set up necessary measures to implement continuous care for patients.
	9 First aids	34	Detect in time sudden changes in health condition of patients/clients.
		35	Give emergency interventions promptly and appropriately.
		36	Coordinate with other health team members effectively.
		37	Perform effective first aid for patients/clients.
	10 Rapport with patients	38	Create trust and confidence for patients/clients, families and team members.
		39	Spend adequate time for communication with client/significant others and team members.
		40	Listen actively to patients/family's concerns, complaints.
	11 Interpersonal communication	41	Identify patients/client's feelings and psychological needs through his/her body language and facial expressions.
		42	Communicate effectively with individuals, families and communities that have limitations in communication due to disease or psychological problems.
		43	Express words, gestures that motivate, encourage and comfort patients/clients.
		44	Demonstrate understanding of culture, belief of patients/clients, families and community when communicate with them.
	12 ICT Skills	45	Utilize audio-visual facilities available to support communication with patients/clients, families and communities.
		46	Utilize effectively and appropriately communication methods with patients/clients, families and communities.
	13 Intelligible explanation	47	Define appropriate information needed to inform to patient/family.
		48	Make psychological preparation for patients/clients and families before inform them the "bad" news.
	14 Health education	49	Collect and analyze information on the needs of health education of individuals, families and communities.
		50	Identify needs and content of information needed to provide to patients, families and communities.
		51	Develop health education plan fitting with socio-cultural features, beliefs of patients, families and communities.
		52	Develop health education materials fitting with patients, families and communities.
		53	Perform health education for individuals, families and groups appropriately and effectively.
		54	Perform evaluation effects of health education and adjust it necessarily.
	15 Teamworking	55	Maintain good relationship with other team members, consider patient as a team member.
		56	Collaborate well with team member in order to make appropriate decisions for quality improvement.
		57	Recommend appropriate intervention to improve client care.
		58	Respect the role and views/opinions of other team members.
		59	Share information with other team members.
		60	Act as representative and advocacy role to protect patients'/clients ' right and safety.
**Domain 2: Management and professional development**			
	16 Documentation	61	Apply principles of management and storage of medical records regulated by the law and by MOH.
		62	Keep information written in medical records confidentially.
		63	Record information in medical record accuracy and timely.
		64	Use collected data about patient's health status as the basis to develop policy and to facilitate care activities provided to patients.
	17 Care management	65	Manage individual tasks and time scientifically and effectively.
		66	Identify tasks or activities need be done based on degree of priority.
		67	Organize, coordinate, delegate tasks for team member scientifically and effectively.
		68	Demonstrate understanding of the relationship between management and utilization of resources effectively to ensure quality and safe care for patients/clients.
		69	Use informatics technology for management, for care of patients and for updated information.
	18 Medical equipment	70	Establish mechanism to manage and operate equipment for patients/clients care and treatment.
		71	Plan for preventive maintenance program.
		72	Operate equipment, facilities used for nursing care to ensure safety, effectiveness and prevent health care associated infections.
	19 Resource management	73	Identify the cost-effectiveness in the utilization of resources.
		74	Develop budget proposal and deploy the plan to use resources for patient's care needs effectively.
	20 Working environment	75	Comply with standards and safety codes prescribed by laws.
		76	Adhere to policies, procedures and protocols on prevention and control of infection.
		77	Comply to rules and regulations regarding care environment. (water, air and noise)
		78	Comply with regulations regarding management and treatment of wastes.
		79	Define steps to follow in case of fire, earthquake and other emergency situations.
		80	Demonstrate understanding on areas related to occupational health and legal documents on safe working environment.
	21 Quality improvement	81	Be aware of the necessity of quality assurance activities, quality improvement through feedback and evaluation of regular practice.
		82	Detect and reports environmental risks in patient care and make appropriate corrective action.
		83	Solicits feedback from patients/clients and significant others regarding care rendered.
		84	Apply proper methods to improve quality of care.
		85	Participate in quality improvement activities in health care centre.
		86	Share with the team member relevant information regarding patients/clients' condition and significant changes in patients/clients' environment.
		87	Review nursing care plan to improve quality of care as well as minimize potential shortcomings.
		88	Make appropriate proposals of means for care and preventions.
		89	Apply scientific evidences in nursing care to improve safety for patients.
	22 Evidence based practice	90	Define and select research areas or research questions that are necessary, reasonable and feasible.
		91	Apply appropriate methods to conduct selected research issues.
		92	Apply appropriate statistical methods to analyze and interpret collected.
		93	Recommend appropriate research result based solutions.
		94	Present research findings to colleagues, patients and related persons.
		95	Utilize research findings in nursing practice to improve quality of nursing practice.
	23 Professional development	96	Identify own learning needs, strengths, weaknesses/ limitations.
		97	Pursue continuing education, participates in formal and non- formal education; Apply learned information for the improvement of care.
		98	Get involved in professional organizations and civic activities.
		99	Project a professional image of the nurse, demonstrate good manners and right conduct at all times.
		100	Possess positive attitude towards change and criticism, listen to suggestions and recommendations, try new strategies or approaches and adapt to changes willingly.
		101	Perform function according to professional standards.
		102	Contribute to improve training and professional development for colleagues.
		103	Contribute to improve the role and status of nursing profession in the health sector and in society.
**Doman 3: Legal and ethics**			
	24 Legal compliance	104	Practice in accordance with the laws related to health, regulations of the Health Ministry and nursing practice.
		105	Compliance with the provisions of the work base.
		106	Implement codes of conduct of the unit/organization and law.
		107	Record and keep care records and documents related to the patient, the health problems of patients in accordance with the standard care practices.
	25 Moral responsibility	108	Accept responsibility and accountability for own decision and care interventions.
		109	Compliance with national and international standards of professional ethics in nursing practice.
		110	Report the violation to the competent agencies and take responsible for that report.

[a] The categories of domains, competencies, and comptency indicators are described based on The Basic Competency Standard for Vietnamese Nurse. (27)
