# Supplementary material for: Effectiveness of a standard clinical training program in new graduate nurses’ competencies in Vietnam: A quasi-experimental longitudinal study with a difference-in-differences design
Source: PLoS One. 2021 Jul 9;16(7):e0254238. doi: 10.1371/journal.pone.0254238 (PMC8270421; doi:10.1371/journal.pone.0254238)
Supplement: S2 File — (PDF) [file pone.0254238.s002.pdf]

Qestionaire for new nurses before training

ID:	□□□□□□□□

Chuyển sang phần câu hỏi

Phần I: Đặc điểm và Thông tin cơ bản

Vui lòng chọn hoặc gõ câu trả lời nếu cần thiết

STT	CÂU HỎI		CÂU TRẢ LỜI	
1	Năm sinh	Năm	Năm	
		Tháng	Tháng	
2	Giới tính	1	Nam	
		2	Nữ	
3	Quê quán	1~63	Tỉnh	
4	Dân tộc	1	Kinh	
		2	Khác (cụ thể:..)	
5	Tình trạng hôn nhân hiện tại?	1	Đã kết hôn	
		2	Chưa kết hôn	
		3	Ly hôn	
		4	Góa vợ/góa chồng	
		5	Khác (cụ thể:..)	
6	Bạn tốt nghiệp trường điều dưỡng thuộc mô hình nào?	1	Công lập	
		2	Tư thục	
7	Trường đào tạo điều dưỡng mà bạn học thuộc tỉnh nào?	1~63	Tỉnh	
		64	Khác (cụ thể:..)	
8	Bạn có bằng cấp gì về điều dưỡng?<br>(Chọn 1 câu trả lời)	1	Trung cấp	
		2	Cao đẳng	
		3	Đại học	
		4	Sau đại học	
		5	Khác (cụ thể:..)	
9	Tháng/năm tốt nghiệp	Năm	Năm	
		Tháng	Tháng	
10	Bạn có bằng cấp gì khác không?	1	Có	
		2	Không	Bỏ qua câu 11 nếu câu 10 trả lời là "Không"
11	Nếu có, bạn có bằng cấp gì?<br>(Chọn 1 câu trả lời)	1	Trung cấp	
		2	Cao đẳng	
		3	Đại học	
		4	Sau đại học	
		5	Khác (cụ thể:..)	
12	Thực tế công việc của bạn hiện nay?	1	Biên chế	
		2	Hợp đồng	
13	Hiện tại bạn làm việc tác tại Khoa/Phòng nào?	1	Nội	
		2	Ngoại	
		3	Sản	
		4	Nhi	
		5	Kiểm soát nhiễm khuẩn	
		6	Cấp cứu	
		7	Khoa khám bệnh	
		8	Phòng mổ	
		9	Xét nghiệm	
		10	Y học cổ truyền	
		11	Hồi sức tích cực	
		12	Khác (cụ thể:..)	
14	Bạn có được (sẽ được) nhận lương/phụ cấp trong thời gian tham gia đào tạo thực hành lâm sàng không ?	1	Có	
		2	Không	Bỏ qua câu 15 nếu câu 14 trả lời là "Không"
15	Nếu có, bạn sẽ nhận được bao nhiêu tiền mỗi tháng trong thời gian tham gia đào tạo thực hành lâm sàng?	1	< 500,000 VND	
		2	501,000~1,000,000 VND	
		3	1,001,000~2,000,000 VND	
		4	2,001,000~ 3,000,000 VND	
		5	3,001,000~ 4,000,000 VND	
		6	4,001,000~ 5,000,000 VND	
		7	5,001,000~ 6,000,000 VND	
		8	6,001,000~ 7,000,000 VND	
		9	> 7,000,000 VND	
		10	Tôi không biết	

16	Bạn có phải nộp học phí khi tham gia đào tạo chương trình đào tạo thực hành lâm sàng không?	1<br>2	Có<br>Không	Bỏ qua câu 17 nếu câu 16 trả lời là "Không"
17	Nếu có, bạn phải nộp học phí là bao nhiêu/tháng?	1<br>2<br>3<br>4<br>5<br>6<br>7<br>8<br>9<br>10	< 500,000 VND<br>500,000~1,000,000 VND<br>1,001,000~2,000,000 VND<br>2,001,000~ 3,000,000 VND<br>3,001,000~ 4,000,000 VND<br>4,001,000~ 5,000,000 VND<br>5,001,000~ 6,000,000 VND<br>6,001,000~ 7,000,000 VND<br>> 7,000,000 VND<br>Tôi không biết	
18	Bạn có được luân khoa trong thời gian học thực hành lâm sàng 9 tháng không?	1<br>2<br>3	Có<br>Không<br>Tôi không biết	

Tiếp tục (Phần II)

Phần II: Mức độ năng lực điều dưỡng

Vui lòng lựa chọn mức độ kỹ năng, kiến thức của bạn tại thời điểm bắt đầu học thực hành lâm sàng theo từng mục với 3 mức (0; 1; 2)
0 : Chưa làm được
1: Làm được dưới sự hướng dẫn/Cần cải thiện
2: Tự làm được

1	Thực hiện đúng các qui định phòng ngừa chuẩn phù hợp với thực tế tại bệnh viện	0<br>1<br>2	Chưa làm được<br>Làm được dưới sự hướng dẫn<br>Tự làm được	
2	Thực hiện đúng các qui định/qui trình phòng ngừa sự cố y khoa	0<br>1<br>2	Chưa làm được<br>Làm được dưới sự hướng dẫn<br>Tự làm được	
3	Thực hiện được các phương pháp kiểm soát đau khi chăm sóc người bệnh và hướng dẫn người bệnh/gia đình NB tham gia kiểm soát đau	0<br>1<br>2	Chưa làm được<br>Làm được dưới sự hướng dẫn<br>Tự làm được	
4	Áp dụng được QTĐD vào lập kế hoạch chăm sóc và thực hiện chăm sóc người bệnh - phù hợp với tình trạng NB và thời điểm chăm sóc	0<br>1<br>2	Chưa làm được<br>Làm được dưới sự hướng dẫn<br>Tự làm được	
5	Thể hiện khả năng giao tiếp phù hợp, tác phong chu đáo, tuan thủ các qui định khi đón tiếp, chuyển viện, xuất viện cho người bệnh	0<br>1<br>2	Chưa làm được<br>Làm được dưới sự hướng dẫn<br>Tự làm được	
6	Nhận định được các dấu hiệu sinh tồn bất thường và đưa ra các quyết định xử lý phù hợp	0<br>1<br>2	Chưa làm được<br>Làm được dưới sự hướng dẫn<br>Tự làm được	
7	Thực hiện các kỹ thuật vệ sinh răng miệng, tắm, gội, thay quần áo đảm bảo đúng quy trình, phù hợp với tình trạng người bệnh	0<br>1<br>2	Chưa làm được<br>Làm được dưới sự hướng dẫn<br>Tự làm được	
8	Thực hiện/ phối hợp thực hiện các kỹ thuật hỗ trợ người bệnh di chuyển: đảm bảo đúng quy trình và an toàn	0<br>1<br>2	Chưa làm được<br>Làm được dưới sự hướng dẫn<br>Tự làm được	
9	Thực hiện các kỹ thuật hỗ trợ người bệnh ăn/uống: đảm bảo đúng quy trình, an toàn và phù hợp với tình trạng người bệnh	0<br>1<br>2	Chưa làm được<br>Làm được dưới sự hướng dẫn<br>Tự làm được	
10	Thực hiện hiệu quả, an toàn, đúng quy trình các kỹ thuật cho người bệnh uống thuốc, tiêm thuốc	0<br>1<br>2	Chưa làm được<br>Làm được dưới sự hướng dẫn<br>Tự làm được	
11	Phát hiện sớm những biểu hiện bất thường trên người bệnh khi dùng thuốc và đưa ra quyết định xử lý phù hợp	0<br>1<br>2	Chưa làm được<br>Làm được dưới sự hướng dẫn<br>Tự làm được	
12	Thực hiện hiệu quả, an toàn, đúng quy trình kỹ thuật truyền dịch, truyền máu cho người bệnh	0<br>1<br>2	Chưa làm được<br>Làm được dưới sự hướng dẫn<br>Tự làm được	
13	Theo dõi được người bệnh trong và sau khi truyền dịch, truyền máu, phát hiện sớm những biểu hiện bất thường trên người bệnh và đưa ra quyết định xử lý phù hợp	0<br>1<br>2	Chưa làm được<br>Làm được dưới sự hướng dẫn<br>Tự làm được	
14	Nhận định được người bệnh cần phải theo dõi lượng dịch vào, ra	0<br>1<br>2	Chưa làm được<br>Làm được dưới sự hướng dẫn<br>Tự làm được	
15	Thực hiện các kỹ thuật chăm sóc, cắt chỉ vết thương trên người bệnh: đúng quy trình, đảm bảo an toàn	0<br>1<br>2	Chưa làm được<br>Làm được dưới sự hướng dẫn<br>Tự làm được	
16	Thực hiện chăm sóc loét tỳ đè độ I, II cho người bệnh theo đúng quy trình kỹ thuật và an toàn	0<br>1<br>2	Chưa làm được<br>Làm được dưới sự hướng dẫn<br>Tự làm được	
17	Thực hiện các kỹ thuật hỗ trợ bài tiết, thông tiểu, dẫn lưu nước tiểu, thụt tháo: đảm bảo đúng quy trình, an toàn và phù hợp với tình trạng người bệnh	0<br>1<br>2	Chưa làm được<br>Làm được dưới sự hướng dẫn<br>Tự làm được	

Qestionaire for new nurses before training

18	Quản lý máy theo dõi dấu hiệu sinh tồn và các thiết bị chăm sóc người bệnh đúng quy định	0	Chưa làm được	
		1	Làm được dưới sự hướng dẫn	
		2	Tự làm được	
19	Thực hiện giao tiếp phù hợp, hiệu quả trong chăm sóc người bệnh (giao tiếp với NB/gia đình NB, với đồng nghiệp; bao gồm cả kỹ năng thông báo tin xấu)	0	Chưa làm được	
		1	Làm được dưới sự hướng dẫn	
		2	Tự làm được	
20	Thể hiện sự hiểu biết về chủ đề tư vấn, giáo dục sức khoẻ; thái độ thân thiện, cởi mở, tôn trọng khi thực hiện tư vấn giáo dục sức khoẻ	0	Chưa làm được	
		1	Làm được dưới sự hướng dẫn	
		2	Tự làm được	
21	Thảo luận các biện pháp tăng cường hiệu quả nhóm	0	Chưa làm được	
		1	Làm được dưới sự hướng dẫn	
		2	Tự làm được	
22	Xác định nhu cầu về sức khỏe và tình trạng sức khỏe của các cá nhân, gia đình và cộng đồng	0	Chưa làm được	
		1	Làm được dưới sự hướng dẫn	
		2	Tự làm được	
23	Giải thích tình trạng sức khỏe của các cá nhân, gia đình và cộng đồng	0	Chưa làm được	
		1	Làm được dưới sự hướng dẫn	
		2	Tự làm được	
24	Thu thập thông tin và phân tích các vấn đề về sức khỏe, bệnh tật để xác định vấn đề về sức khỏe và bệnh tật của cá nhân, gia đình và cộng đồng	0	Chưa làm được	
		1	Làm được dưới sự hướng dẫn	
		2	Tự làm được	
25	Ra các quyết định về chăm sóc cho người bệnh, gia đình và cộng đồng an toàn và hiệu quả	0	Chưa làm được	
		1	Làm được dưới sự hướng dẫn	
		2	Tự làm được	
26	Cung cấp dịch vụ chăm sóc phù hợp văn hóa, tín ngưỡng	0	Chưa làm được	
		1	Làm được dưới sự hướng dẫn	
		2	Tự làm được	
27	Theo dõi quan sát sự tiến triển của các can thiệp điều dưỡng	0	Chưa làm được	
		1	Làm được dưới sự hướng dẫn	
		2	Tự làm được	
28	Phân tích và xác định được những nhu cầu chăm sóc ưu tiên của cá nhân, gia đình và cộng đồng	0	Chưa làm được	
		1	Làm được dưới sự hướng dẫn	
		2	Tự làm được	
29	Thực hiện các can thiệp chăm sóc đáp ứng nhu cầu chăm sóc ưu tiên của cá nhân, gia đình và cộng đồng	0	Chưa làm được	
		1	Làm được dưới sự hướng dẫn	
		2	Tự làm được	
30	Thực hiện nhận định người bệnh toàn diện và có hệ thống	0	Chưa làm được	
		1	Làm được dưới sự hướng dẫn	
		2	Tự làm được	
31	Tập hợp và ghi đầy đủ thông tin thích hợp vào hồ sơ điều dưỡng	0	Chưa làm được	
		1	Làm được dưới sự hướng dẫn	
		2	Tự làm được	
32	Phân tích và diễn giải các thông tin về người bệnh một cách chính xác, lập kế hoạch chăm sóc.	0	Chưa làm được	
		1	Làm được dưới sự hướng dẫn	
		2	Tự làm được	
33	Lập kế hoạch chăm sóc điều dưỡng dựa trên nhận định người bệnh và có sự thống nhất với đồng nghiệp, người nhà người bệnh về các vấn đề ưu tiên, sự mong muốn và kết quả mong đợi cho người bệnh	0	Chưa làm được	
		1	Làm được dưới sự hướng dẫn	
		2	Tự làm được	
34	Giải thích các can thiệp điều dưỡng cho người bệnh, gia đình người bệnh và thực hiện chăm sóc điều dưỡng, bảo đảm an toàn, thoải mái, hiệu quả cho người bệnh	0	Chưa làm được	
		1	Làm được dưới sự hướng dẫn	
		2	Tự làm được	
35	Hướng dẫn người bệnh, gia đình người bệnh các phương pháp tự chăm sóc một cách phù hợp	0	Chưa làm được	
		1	Làm được dưới sự hướng dẫn	
		2	Tự làm được	
36	Đánh giá quá trình chăm sóc và điều chỉnh kế hoạch chăm sóc	0	Chưa làm được	
		1	Làm được dưới sự hướng dẫn	
		2	Tự làm được	
37	Thực hiện các công việc cần thiết để hỗ trợ người bệnh xuất viện	0	Chưa làm được	
		1	Làm được dưới sự hướng dẫn	
		2	Tự làm được	
38	Tuyên truyền giáo dục sức khỏe	0	Chưa làm được	
		1	Làm được dưới sự hướng dẫn	
		2	Tự làm được	
39	Thực hiện các biện pháp an toàn	0	Chưa làm được	
		1	Làm được dưới sự hướng dẫn	
		2	Tự làm được	
40	Tạo môi trường chăm sóc	0	Chưa làm được	
		1	Làm được dưới sự hướng dẫn	
		2	Tự làm được	
41	Đảm bảo sự riêng tư cá nhân	0	Chưa làm được	
		1	Làm được dưới sự hướng dẫn	
		2	Tự làm được	
42	Tuân thủ các bước của quy trình điều dưỡng	0	Chưa làm được	
		1	Làm được dưới sự hướng dẫn	
		2	Tự làm được	
43	Thực hiện thành thạo kỹ thuật điều dưỡng trong phạm vi chuyên môn	0	Chưa làm được	
		1	Làm được dưới sự hướng dẫn	
		2	Tự làm được	
44	Tuân thủ các quy định về vô khuẩn và kiểm soát nhiễm khuẩn	0	Chưa làm được	
		1	Làm được dưới sự hướng dẫn	
		2	Tự làm được	
		0	Chưa làm được	

45	Khai thác tiền sử dị ứng thuốc của người bệnh	1<br>2	Làm được dưới sự hướng dẫn<br>Tự làm được	
46	Tuân thủ quy tắc khi dùng thuốc	0<br>1<br>2	Chưa làm được<br>Làm được dưới sự hướng dẫn<br>Tự làm được	
47	Hướng dẫn người bệnh dùng thuốc đúng và an toàn	0<br>1<br>2	Chưa làm được<br>Làm được dưới sự hướng dẫn<br>Tự làm được	
48	Phát hiện và xử trí ban đầu các dấu hiệu của phản ứng có hại của thuốc	0<br>1<br>2	Chưa làm được<br>Làm được dưới sự hướng dẫn<br>Tự làm được	
49	Nhận biết sự tương tác giữa thuốc với thuốc và thuốc với thức ăn	0<br>1<br>2	Chưa làm được<br>Làm được dưới sự hướng dẫn<br>Tự làm được	
50	Đánh giá hiệu quả của việc dùng thuốc	0<br>1<br>2	Chưa làm được<br>Làm được dưới sự hướng dẫn<br>Tự làm được	
51	Ghi chép và công khai việc sử dụng thuốc cho người bệnh	0<br>1<br>2	Chưa làm được<br>Làm được dưới sự hướng dẫn<br>Tự làm được	
52	Bàn giao tình trạng của người bệnh với nhóm chăm sóc kế tiếp một cách cụ thể, đầy đủ và chính xác	0<br>1<br>2	Chưa làm được<br>Làm được dưới sự hướng dẫn<br>Tự làm được	
53	Phối hợp hiệu quả với người bệnh, gia đình và đồng nghiệp để đảm bảo chăm sóc liên tục cho người bệnh	0<br>1<br>2	Chưa làm được<br>Làm được dưới sự hướng dẫn<br>Tự làm được	
54	Thiết lập các biện pháp để thực hiện chăm sóc liên tục cho người bệnh	0<br>1<br>2	Chưa làm được<br>Làm được dưới sự hướng dẫn<br>Tự làm được	
55	Phát hiện sớm những thay đổi đột ngột về tình trạng sức khỏe người bệnh	0<br>1<br>2	Chưa làm được<br>Làm được dưới sự hướng dẫn<br>Tự làm được	
56	Ra quyết định xử trí sơ cứu, cấp cứu kịp thời và phù hợp	0<br>1<br>2	Chưa làm được<br>Làm được dưới sự hướng dẫn<br>Tự làm được	
57	Phối hợp hiệu quả với các thành viên nhóm chăm sóc trong sơ cứu, cấp cứu	0<br>1<br>2	Chưa làm được<br>Làm được dưới sự hướng dẫn<br>Tự làm được	
58	Thực hiện sơ cứu, cấp cứu hiệu quả cho người bệnh	0<br>1<br>2	Chưa làm được<br>Làm được dưới sự hướng dẫn<br>Tự làm được	
59	Tạo dựng niềm tin đối với người bệnh, người nhà và thành viên trong nhóm chăm sóc	0<br>1<br>2	Chưa làm được<br>Làm được dưới sự hướng dẫn<br>Tự làm được	
60	Dành thời gian cần thiết để giao tiếp với người bệnh, người nhà và thành viên trong nhóm chăm sóc	0<br>1<br>2	Chưa làm được<br>Làm được dưới sự hướng dẫn<br>Tự làm được	
61	Lắng nghe và đáp ứng thích hợp những băn khoăn, lo lắng của người bệnh và người nhà người bệnh	0<br>1<br>2	Chưa làm được<br>Làm được dưới sự hướng dẫn<br>Tự làm được	
62	Nhận biết tâm lý và nhu cầu của người bệnh qua những biểu hiện nét mặt và ngôn ngữ cơ thể của người bệnh	0<br>1<br>2	Chưa làm được<br>Làm được dưới sự hướng dẫn<br>Tự làm được	
63	Giao tiếp hiệu quả với các cá nhân, gia đình, cộng đồng có các trở ngại về giao tiếp do bệnh tật, do những kh ó khăn về tâm lý	0<br>1<br>2	Chưa làm được<br>Làm được dưới sự hướng dẫn<br>Tự làm được	
64	Thể hiện lời nói, cử chỉ động viên, khuyến khích người bệnh an tâm điều trị	0<br>1<br>2	Chưa làm được<br>Làm được dưới sự hướng dẫn<br>Tự làm được	
65	Thể hiện sự hiểu biết về văn hóa, tín ngưỡng trong giao tiếp với người bệnh, gia đình và nhóm người	0<br>1<br>2	Chưa làm được<br>Làm được dưới sự hướng dẫn<br>Tự làm được	
66	Sử dụng các phương tiện nghe nhìn sẵn có để truyền thông và hỗ trợ giao tiếp với người bệnh, người nhà v à cộng đồng	0<br>1<br>2	Chưa làm được<br>Làm được dưới sự hướng dẫn<br>Tự làm được	
67	Sử dụng các phương pháp, hình thức giao tiếp hiệu quả và thích hợp với người bệnh, người nhà người bệnh	0<br>1<br>2	Chưa làm được<br>Làm được dưới sự hướng dẫn<br>Tự làm được	
68	Xác định những thông tin cần cung cấp cho người bệnh và gia đình	0<br>1<br>2	Chưa làm được<br>Làm được dưới sự hướng dẫn<br>Tự làm được	
69	Chuẩn bị về tâm lý cho người bệnh và gia đình trước khi cung cấp những thông tin "xấu"	0<br>1<br>2	Chưa làm được<br>Làm được dưới sự hướng dẫn<br>Tự làm được	
70	Thu thập và phân tích thông tin về nhu cầu hiểu biết của cá nhân, gia đình, và cộng đồng về hướng dẫn, giá o dục sức khỏe	0<br>1<br>2	Chưa làm được<br>Làm được dưới sự hướng dẫn<br>Tự làm được	
71	Xác định nhu cầu và những nội dung cần hướng dẫn, giáo dục sức khỏe cho cá nhân, gia đình và cộng đồng	0<br>1<br>2	Chưa làm được<br>Làm được dưới sự hướng dẫn<br>Tự làm được	
72	Xây dựng kế hoạch giáo dục sức khỏe phù hợp với văn hóa, xã hội và tín ngưỡng của cá nhân, gia đình và cộng đồng	0<br>1	Chưa làm được<br>Làm được dưới sự hướng dẫn	

	cộng đồng	2	Tự làm được	
73	Xây dựng tài liệu giáo dục sức khỏe phù hợp với trình độ của đối tượng	0	Chưa làm được	
		1	Làm được dưới sự hướng dẫn	
		2	Tự làm được	
74	Thực hiện tư vấn, truyền thông giáo dục sức khỏe phù hợp, hiệu quả	0	Chưa làm được	
		1	Làm được dưới sự hướng dẫn	
		2	Tự làm được	
75	Đánh giá kết quả giáo dục sức khỏe và điều chỉnh kế hoạch giáo dục sức khỏe dựa trên mục tiêu và kết quả mong chờ	0	Chưa làm được	
		1	Làm được dưới sự hướng dẫn	
		2	Tự làm được	
76	Duy trì tốt mối quan hệ với các thành viên trong nhóm, coi người bệnh như một cộng sự trong nhóm chăm sóc	0	Chưa làm được	
		1	Làm được dưới sự hướng dẫn	
		2	Tự làm được	
77	Hợp tác tốt với các thành viên trong nhóm chăm sóc để đưa ra các quyết định phù hợp nhằm cải thiện chất lượng chăm sóc	0	Chưa làm được	
		1	Làm được dưới sự hướng dẫn	
		2	Tự làm được	
78	Hợp tác tốt với các thành viên nhóm chăm sóc trong việc theo dõi, chăm sóc, điều trị người bệnh và thực hiện nhiệm vụ được giao	0	Chưa làm được	
		1	Làm được dưới sự hướng dẫn	
		2	Tự làm được	
79	Tôn trọng vai trò và quan điểm của đồng nghiệp	0	Chưa làm được	
		1	Làm được dưới sự hướng dẫn	
		2	Tự làm được	
80	Chia sẻ thông tin một cách hiệu quả với các thành viên trong nhóm chăm sóc	0	Chưa làm được	
		1	Làm được dưới sự hướng dẫn	
		2	Tự làm được	
81	Thực hiện vai trò đại diện hoặc biện hộ cho người bệnh để bảo đảm các quyền, lợi ích và vì sự an toàn của người bệnh	0	Chưa làm được	
		1	Làm được dưới sự hướng dẫn	
		2	Tự làm được	
82	Thực hiện các quy chế quản lý, lưu giữ hồ sơ bệnh án theo quy định luật pháp và của Bộ Y tế	0	Chưa làm được	
		1	Làm được dưới sự hướng dẫn	
		2	Tự làm được	
83	Bảo mật thông tin trong hồ sơ bệnh án và Phiếu chăm sóc của người bệnh	0	Chưa làm được	
		1	Làm được dưới sự hướng dẫn	
		2	Tự làm được	
84	Ghi chép hồ sơ điều dưỡng bảo đảm tính khách quan, chính xác, đầy đủ và kịp thời	0	Chưa làm được	
		1	Làm được dưới sự hướng dẫn	
		2	Tự làm được	
85	Sử dụng các dữ liệu thu thập được về tình trạng sức khỏe người bệnh làm cơ sở để xây dựng chính sách và tạo thuận lợi cho việc chăm sóc người bệnh	0	Chưa làm được	
		1	Làm được dưới sự hướng dẫn	
		2	Tự làm được	
86	Quản lý công việc, thời gian của cá nhân hiệu quả và khoa học	0	Chưa làm được	
		1	Làm được dưới sự hướng dẫn	
		2	Tự làm được	
87	Xác định các công việc hoặc nhiệm vụ cần hoàn thành theo thứ tự ưu tiên	0	Chưa làm được	
		1	Làm được dưới sự hướng dẫn	
		2	Tự làm được	
88	Tổ chức, điều phối, phân công và ủy quyền nhiệm vụ cho các thành viên của nhóm chăm sóc một cách khoa học, hợp lý và hiệu quả	0	Chưa làm được	
		1	Làm được dưới sự hướng dẫn	
		2	Tự làm được	
89	Thể hiện sự hiểu biết về mối quan hệ giữa quản lý và sử dụng các nguồn lực có hiệu quả để đảm bảo chất lượng chăm sóc và an toàn cho người bệnh	0	Chưa làm được	
		1	Làm được dưới sự hướng dẫn	
		2	Tự làm được	
90	Sử dụng công nghệ thông tin trong quản lý và chăm sóc người bệnh cũng như cập nhật kiến thức chuyên m ôn	0	Chưa làm được	
		1	Làm được dưới sự hướng dẫn	
		2	Tự làm được	
91	Thiết lập các cơ chế quản lý, phát huy tối đa chức năng hoạt động của các phương tiện, trang thiết bị phục vụ cho chăm sóc và điều trị	0	Chưa làm được	
		1	Làm được dưới sự hướng dẫn	
		2	Tự làm được	
92	Lập và thực hiện kế hoạch bảo trì phương tiện, trang thiết bị do mình phụ trách	0	Chưa làm được	
		1	Làm được dưới sự hướng dẫn	
		2	Tự làm được	
93	Vận hành các trang thiết bị, phương tiện sử dụng trong chăm sóc bảo đảm an toàn, hiệu quả và phòng trá nh nhiễm khuẩn liên quan đến chăm sóc y tế	0	Chưa làm được	
		1	Làm được dưới sự hướng dẫn	
		2	Tự làm được	
94	Nhận biết được hiệu quả kinh tế khi sử dụng các nguồn lực sẵn có tại nơi làm việc để sử dụng thích hợp, hiệu quả	0	Chưa làm được	
		1	Làm được dưới sự hướng dẫn	
		2	Tự làm được	
95	Xây dựng và thực hiện kế hoạch sử dụng các nguồn lực trong chăm sóc người bệnh thuộc phạm vi phân cô ng hiệu quả	0	Chưa làm được	
		1	Làm được dưới sự hướng dẫn	
		2	Tự làm được	
96	Tuân thủ các tiêu chuẩn và quy tắc về an toàn lao động	0	Chưa làm được	
		1	Làm được dưới sự hướng dẫn	
		2	Tự làm được	
97	Tuân thủ các chính sách, quy trình về phòng ngừa cách ly và kiểm soát nhiễm khuẩn	0	Chưa làm được	
		1	Làm được dưới sự hướng dẫn	
		2	Tự làm được	
98	Tuân thủ các quy định về kiểm soát môi trường chăm sóc (tiếng ồn, không khí, nguồn nước…)	0	Chưa làm được	
		1	Làm được dưới sự hướng dẫn	
		2	Tự làm được	
99	Tuân thủ quy định về quản lý, xử lý chất thải	0	Chưa làm được	
		1	Làm được dưới sự hướng dẫn	
		2	Tự làm được	

Qestionaire for new nurses before training

100	Tuân thủ các bước về an toàn phòng cháy chữa cháy, động đất hoặc các trường hợp khẩn cấp khác	0<br>1<br>2	Chưa làm được<br>Làm được dưới sự hướng dẫn<br>Tự làm được	
101	Thể hiện sự hiểu biết về những khía cạnh có liên quan đến sức khỏe nghề nghiệp và luật pháp về an toàn lao động	0<br>1<br>2	Chưa làm được<br>Làm được dưới sự hướng dẫn<br>Tự làm được	
102	Nhận thức được sự cần thiết về các hoạt động bảo đảm chất lượng, cải tiến chất lượng thông qua sự nghiên cứu, phản hồi và đánh giá thực hành thường xuyên	0<br>1<br>2	Chưa làm được<br>Làm được dưới sự hướng dẫn<br>Tự làm được	
103	Phát hiện, báo cáo và đưa ra các hành động khắc phục phù hợp các nguy cơ trong môi trường chăm sóc người bệnh	0<br>1<br>2	Chưa làm được<br>Làm được dưới sự hướng dẫn<br>Tự làm được	
104	Nhận phản hồi từ người bệnh, gia đình và các đối tượng liên quan để cải tiến chất lượng các hoạt động chăm sóc	0<br>1<br>2	Chưa làm được<br>Làm được dưới sự hướng dẫn<br>Tự làm được	
105	Áp dụng các phương pháp cải tiến chất lượng phù hợp	0<br>1<br>2	Chưa làm được<br>Làm được dưới sự hướng dẫn<br>Tự làm được	
106	Tham gia các hoạt động cải tiến chất lượng tại cơ sở	0<br>1<br>2	Chưa làm được<br>Làm được dưới sự hướng dẫn<br>Tự làm được	
107	Chia sẻ các thông tin liên quan đến tình trạng người bệnh với các thành viên trong nhóm chăm sóc	0<br>1<br>2	Chưa làm được<br>Làm được dưới sự hướng dẫn<br>Tự làm được	
108	Bình phiếu chăm sóc để cải tiến và khắc phục những tồn tại về chuyên môn và thủ tục hành chính	0<br>1<br>2	Chưa làm được<br>Làm được dưới sự hướng dẫn<br>Tự làm được	
109	Đưa ra những đề xuất phù hợp về các biện pháp chăm sóc và phòng ngừa bệnh	0<br>1<br>2	Chưa làm được<br>Làm được dưới sự hướng dẫn<br>Tự làm được	
110	Sử dụng bằng chứng áp dụng vào thực hành chăm sóc để tăng cường sự an toàn trong chăm sóc người bệnh	0<br>1<br>2	Chưa làm được<br>Làm được dưới sự hướng dẫn<br>Tự làm được	
111	Xác định và lựa chọn các lĩnh vực và vấn đề nghiên cứu phù hợp, cần thiết và khả thi	0<br>1<br>2	Chưa làm được<br>Làm được dưới sự hướng dẫn<br>Tự làm được	
112	Áp dụng các phương pháp phù hợp để tiến hành nghiên cứu những vấn đề đã lựa chọn	0<br>1<br>2	Chưa làm được<br>Làm được dưới sự hướng dẫn<br>Tự làm được	
113	Sử dụng phương pháp thống kê thích hợp để phân tích và diễn giải dữ liệu thu thập được	0<br>1<br>2	Chưa làm được<br>Làm được dưới sự hướng dẫn<br>Tự làm được	
114	Đề xuất các giải pháp thích hợp dựa trên kết quả nghiên cứu	0<br>1<br>2	Chưa làm được<br>Làm được dưới sự hướng dẫn<br>Tự làm được	
115	Trình bày, chia sẻ kết quả nghiên cứu với đồng nghiệp, người bệnh và những người có liên quan	0<br>1<br>2	Chưa làm được<br>Làm được dưới sự hướng dẫn<br>Tự làm được	
116	Ứng dụng kết quả nghiên cứu khoa học vào thực hành điều dưỡng. Sử dụng các bằng chứng từ nghiên cứu khoa học để nâng cao chất lượng thực hành chăm sóc điều dưỡng	0<br>1<br>2	Chưa làm được<br>Làm được dưới sự hướng dẫn<br>Tự làm được	
117	Xác định rõ mục tiêu, nguyện vọng phát triển nghề nghiệp, nhu cầu học tập, điểm mạnh, điểm yếu của bản thân	0<br>1<br>2	Chưa làm được<br>Làm được dưới sự hướng dẫn<br>Tự làm được	
118	Học tập liên tục để cập nhật kiến thức, kỹ năng và ứng dụng kiến thức đã học để nâng cao chất lượng thực hành chăm sóc điều dưỡng	0<br>1<br>2	Chưa làm được<br>Làm được dưới sự hướng dẫn<br>Tự làm được	
119	Tham gia vào các hoạt động của tổ chức nghề nghiệp	0<br>1<br>2	Chưa làm được<br>Làm được dưới sự hướng dẫn<br>Tự làm được	
120	Quảng bá hình ảnh của người điều dưỡng, thể hiện tác phong và tư cách tốt, trang phục phù hợp, lời nói thuyết phục và cách cư xử đúng mực	0<br>1<br>2	Chưa làm được<br>Làm được dưới sự hướng dẫn<br>Tự làm được	
121	Thể hiện thái độ tích cực với những đổi mới và những quan điểm trái chiều, thể hiện sự lắng nghe các kiến nghị và đề xuất, thử nghiệm những phương pháp mới và thích nghi với những thay đổi	0<br>1<br>2	Chưa làm được<br>Làm được dưới sự hướng dẫn<br>Tự làm được	
122	Thực hiện chăm sóc theo các tiêu chuẩn thực hành điều dưỡng	0<br>1<br>2	Chưa làm được<br>Làm được dưới sự hướng dẫn<br>Tự làm được	
123	Đóng góp vào việc đào tạo nâng cao trình độ và phát triển nghề nghiệp cho đồng nghiệp	0<br>1<br>2	Chưa làm được<br>Làm được dưới sự hướng dẫn<br>Tự làm được	
124	Đóng góp vào việc nâng cao vai trò, vị thế của người điều dưỡng, ngành điều dưỡng trong ngành y tế và trong xã hội	0<br>1<br>2	Chưa làm được<br>Làm được dưới sự hướng dẫn<br>Tự làm được	
125	Hành nghề theo quy định của pháp luật liên quan đến y tế, quy định của Bộ Y tế và thực hành điều dưỡng	0<br>1<br>2	Chưa làm được<br>Làm được dưới sự hướng dẫn<br>Tự làm được	
126	Tuân thủ các quy định của cơ sở nơi làm việc	0<br>1<br>2	Chưa làm được<br>Làm được dưới sự hướng dẫn<br>Tự làm được	
		0	Chưa làm được	

127	Thực hiện tốt quy tắc ứng xử của đơn vị/tổ chức và luật định.	1	Làm được dưới sự hướng dẫn	
		2	Tự làm được	
128	Ghi chép và bảo quản hồ sơ chăm sóc và tài liệu liên quan đến người bệnh, các vấn đề sức khỏe của người bệnh phù hợp với các tiêu chuẩn thực hành chăm sóc	0	Chưa làm được	
		1	Làm được dưới sự hướng dẫn	
		2	Tự làm được	
129	Chịu trách nhiệm cá nhân khi đưa ra các quyết định và can thiệp chăm sóc	0	Chưa làm được	
		1	Làm được dưới sự hướng dẫn	
		2	Tự làm được	
130	Tuân thủ Tiêu chuẩn đạo đức nghề nghiệp của quốc gia và quốc tế trong thực hành điều dưỡng	0	Chưa làm được	
		1	Làm được dưới sự hướng dẫn	
		2	Tự làm được	
131	Báo cáo các hành vi vi phạm với cơ quan có thẩm quyền và chịu trách nhiệm cá nhân với báo cáo đó	0	Chưa làm được	
		1	Làm được dưới sự hướng dẫn	
		2	Tự làm được	

Hoàn thành

ID:	□□□□□□□□

Go to Questionnaire

Section Ⅰ: Basic Characteristics & Information

Please select answer or type answer if necessary

No of Q	QUESTION		ANSWER	
1	Year of birth	Year	Year:	
		Month	Month:	
2	Gender	1	Male	
		2	Female	
3	Where do you come from (hometown)?	1~63	Province:	
4	Ethnic group	1	Kinh	
		2	Other (specify:..)	
5	Which is your current marital status?	1	Married	
		2	Never married	
		3	Divorced	
		4	Widowed	
		5	Other (specify:..)	
6	What kind of nursing school did you graduate?	1	Public	
		2	Private	
7	Which province your nursing training insitituiton belong to?	1~63	Province:	
		64	other (specify:..)	
8	Which nursing training program have you graduated?<br>(One choice is applicable)	1	Intermidiate	
		2	Collage	
		3	University	
		4	Post graduate (Master course)	
		6	Other (specify:..)	
9	Month and year of graduation	Year	Year:	
		Month	Month:	
10	Do you have any other degree?	1	yes	
		2	no	Skip Q11 if Q10 is "2. No"
11	If yes, what degree do you have?<br>(One choice is applicable)	1	Intermidiate	
		2	Collage	
		3	University	
		4	Post graduate (Master course)	
		5	Other (specify:..)	
12	Which is your current working status ?	1	permanent staff	
		2	contract	
13	Currenlty, which division/department are you working?	1	Internal medicine	
		2	Surgery	
		3	Obstetrics	
		4	Peadiatrics	
		5	Infection	
		6	Emergency	
		7	Out patient	
		8	Operation room	
		9	Testing	
		10	Traditional Medicine	
		11	ICU	
		12	Other (specify…................................)	
14	Do you(Are you supposed to) receive salary during clinical training ?	1	yes	
		2	no	Skip Q14 if Q13 is "2. No"

15	If yes, how much do you (are supposed to) receive monthly salary during clinical training ?	1<br>2<br>3<br>4<br>5<br>6<br>7<br>8<br>9<br>10	< 500,000 VND<br>501,000~1,000,000 VND<br>1,001,000~2,000,000 VND<br>2,001,000~ 3,000,000 VND<br>3,001,000~ 4,000,000 VND<br>4,001,000~ 5,000,000 VND<br>5,001,000~ 6,000,000 VND<br>6,001,000~ 7,000,000 VND<br>> 7,000,000 VND<br>I don't know	
16	Do you have to pay tution fee for clinical training ?	1<br>2	yes<br>no	 Skip Q16 if Q15 is “2. No”
17	If yes, how much do you have to pay as tution fee per month ?	1<br>2<br>3<br>4<br>5<br>6<br>7<br>8<br>9<br>10	< 500,000 VND<br>501,000~1,000,000 VND<br>1,001,000~2,000,000 VND<br>2,001,000~ 3,000,000 VND<br>3,001,000~ 4,000,000 VND<br>4,001,000~ 5,000,000 VND<br>5,001,000~ 6,000,000 VND<br>6,001,000~ 7,000,000 VND<br>> 7,000,000 VND<br>I don't know	
18	Will you rotate to different division during coming 9 month-trainig program?	1<br>2<br>3	yes<br>no<br>98. I don’t know	

GO TO next page (Section II)

Section Ⅱ: competency level of nursing

Please kindly select your skill or knowledge level at the beginning of Clinical Training at each item by 3 levels (0; 1; 2)

0 : Cannot do

1: Can do with support/need to be improved

2: Can do independently

1	Perform correctly the standard prevention regulations suitable with reality of the hospital	0<br>1<br>2	Cannot do<br>Can do with support<br>Can do independently	
2	Properly perform regulations/procedure of medical incidents prevention	0<br>1<br>2	Cannot do<br>Can do with support<br>Can do independently	
3	Perform pain management methods for patient care and provide guidance of pain management to patients/families	0<br>1<br>2	Cannot do<br>Can do with support<br>Can do independently	
4	Apply nursing process in making care plan and performing patient care at clinical departments – suitable with patient status and care timing	0<br>1<br>2	Cannot do<br>Can do with support<br>Can do independently	
5	Demonstrate appropriate communication skills, attentive behavior, complying with regulations when receiving, transferring and discharging patients	0<br>1<br>2	Cannot do<br>Can do with support<br>Can do independently	
6	Assess abnormal vital signs and make suitable decision for dealing with	0<br>1<br>2	Cannot do<br>Can do with support<br>Can do independently	
7	Perform techniques of oral hygiene, bath, shampoo, dress change to ensure proper procedure and suitable with patient’s conditions	0<br>1<br>2	Cannot do<br>Can do with support<br>Can do independently	
8	Perform/cooperative perform patient movement supportive techniques: ensuring proper procedure and safe.	0<br>1<br>2	Cannot do<br>Can do with support<br>Can do independently	
9	Practice supporting the patient to eat/drink: secure the procedure, safety and correspondence with patient’s clinical condition	0<br>1<br>2	Cannot do<br>Can do with support<br>Can do independently	
10	Effectively, safely and properly perform the techniques of giving medication, including injection to patients	0<br>1<br>2	Cannot do<br>Can do with support<br>Can do independently	
11	Early detection of abnormal manifestations on patients when taking drugs and making appropriate treatment decisions	0<br>1<br>2	Cannot do<br>Can do with support<br>Can do independently	
12	Perform effectively, safely, properly technical procedure of fluid infusion, blood transfusion in patients	0<br>1<br>2	Cannot do<br>Can do with support<br>Can do independently	
13	Monitor patient during and after fluid, blood transfusion, to detect early abnormal manifestations of patient and give decision for suitable management	0<br>1<br>2	Cannot do<br>Can do with support<br>Can do independently	
14	Perform correctly technical procedure of in-out fluid monitoring	0<br>1<br>2	Cannot do<br>Can do with support<br>Can do independently	

Qestionaire for new nurses before training

15	Implement effectively and safely, properly nursing care of injury and drainage tube	0<br>1<br>2	Cannot do<br>Can do with support<br>Can do independently	
16	Perform pressure ulcer care of level I, II to patient in accordance with technical procedure and safe	0<br>1<br>2	Cannot do<br>Can do with support<br>Can do independently	
17	Perform techniques for excretion support, urinary catheterization, urinary drainage, enema: ensure procedure compliance, safety and suitable with patient's conditions	0<br>1<br>2	Cannot do<br>Can do with support<br>Can do independently	
18	Manage vital sign monitors and patient care equipments in accordance with regulation	0<br>1<br>2	Cannot do<br>Can do with support<br>Can do independently	
19	Perform appropriate and effective communication in patient care (communicate with patient, patient's family, colleague including skills of bad news notification	0<br>1<br>2	Cannot do<br>Can do with support<br>Can do independently	
20	Have good knowledge about health consultation and health education; with friendly attitude, open-hearted and respectful performance during health educating period	0<br>1<br>2	Cannot do<br>Can do with support<br>Can do independently	
21	Discuss measures to enhance team work effectiveness	0<br>1<br>2	Cannot do<br>Can do with support<br>Can do independently	
22	Identifies the health care need of the individuals and groups	0<br>1<br>2	Cannot do<br>Can do with support<br>Can do independently	
23	Explains the health status of the individuals, groups, and communities	0<br>1<br>2	Cannot do<br>Can do with support<br>Can do independently	
24	Collects the information and analyzes to identify health problems of the individuals, families and communities	0<br>1<br>2	Cannot do<br>Can do with support<br>Can do independently	
25	Selects proper nursing care activities to each patients, families and communities	0<br>1<br>2	Cannot do<br>Can do with support<br>Can do independently	
26	provides proper nursing service to culture, belief	0<br>1<br>2	Cannot do<br>Can do with support<br>Can do independently	
27	Monitors the improvement of nursing interventions	0<br>1<br>2	Cannot do<br>Can do with support<br>Can do independently	
28	Identifies and analyses the priority care needs of patients, families and communities	0<br>1<br>2	Cannot do<br>Can do with support<br>Can do independently	
29	Performs the care intervention to meet the priority care needs of patients, families and communities	0<br>1<br>2	Cannot do<br>Can do with support<br>Can do independently	
30	Performs patient identification comprehensively and systematically	0<br>1<br>2	Cannot do<br>Can do with support<br>Can do independently	
31	Collects and fully fills appropriate information in nursing profile	0<br>1<br>2	Cannot do<br>Can do with support<br>Can do independently	
32	Analyses and explains the patient's information exactly.	0<br>1<br>2	Cannot do<br>Can do with support<br>Can do independently	
33	makes nursing care plan based on patient identification and in collaboration with colleagues, patient's family about priority issues, expectations and patient's expected results.	0<br>1<br>2	Cannot do<br>Can do with support<br>Can do independently	
34	Explains the nursing interventions to patients, families and performs nursing intervention following the nursing care plan in a safety, effectively and timely manner.	0<br>1<br>2	Cannot do<br>Can do with support<br>Can do independently	
35	Provides guides to patients, families the appropriate self-care methods	0<br>1<br>2	Cannot do<br>Can do with support<br>Can do independently	
36	Evaluate the nursing care process and revises the care plan based on the patient's health condition and expected results.	0<br>1<br>2	Cannot do<br>Can do with support<br>Can do independently	
37	Performs necessary activities to support clients who is going to discharge	0<br>1<br>2	Cannot do<br>Can do with support<br>Can do independently	
38	Provides health educations and guides preventions to the patient	0<br>1<br>2	Cannot do<br>Can do with support<br>Can do independently	
39	Performs safety measures in patient care	0<br>1<br>2	Cannot do<br>Can do with support<br>Can do independently	
40	Performs comfort caring environment in patients care	0<br>1<br>2	Cannot do<br>Can do with support<br>Can do independently	

Qestionaire for new nurses before training

41	Ensures the privacy in patient care	0<br>1<br>2	Cannot do<br>Can do with support<br>Can do independently	
42	follows steps of nursing process	0<br>1<br>2	Cannot do<br>Can do with support<br>Can do independently	
43	Performs correctly nursing techniques in the professional scope	0<br>1<br>2	Cannot do<br>Can do with support<br>Can do independently	
44	Follows the regulations on sterilization and infection control	0<br>1<br>2	Cannot do<br>Can do with support<br>Can do independently	
45	Exploits history of drug allergy of patients	0<br>1<br>2	Cannot do<br>Can do with support<br>Can do independently	
46	Follows the rules of medications	0<br>1<br>2	Cannot do<br>Can do with support<br>Can do independently	
47	Provides guidance of drug usage correctly and safety.	0<br>1<br>2	Cannot do<br>Can do with support<br>Can do independently	
48	finds out and initially treats any drug allergy	0<br>1<br>2	Cannot do<br>Can do with support<br>Can do independently	
49	Be aware of potential drug – drug or drug-food interactions	0<br>1<br>2	Cannot do<br>Can do with support<br>Can do independently	
50	Evaluates effectiveness of drug usage	0<br>1<br>2	Cannot do<br>Can do with support<br>Can do independently	
51	Records and publicizes drug usage to the patient	0<br>1<br>2	Cannot do<br>Can do with support<br>Can do independently	
52	Hands over the patient's status to the next care team specifically and accurately	0<br>1<br>2	Cannot do<br>Can do with support<br>Can do independently	
53	Effectively cooperates with patient, families and colleagues to ensure patient continuous cares	0<br>1<br>2	Cannot do<br>Can do with support<br>Can do independently	
54	Establishes measures to perform continuous cares to the patients	0<br>1<br>2	Cannot do<br>Can do with support<br>Can do independently	
55	Early finds out sudden changes in health condition of patients	0<br>1<br>2	Cannot do<br>Can do with support<br>Can do independently	
56	Decides timely and properly first aid, emergency treatment	0<br>1<br>2	Cannot do<br>Can do with support<br>Can do independently	
57	Effectively cooperates with care team members in first aid, emergency	0<br>1<br>2	Cannot do<br>Can do with support<br>Can do independently	
58	Performs effectively first aid, emergency to the patients	0<br>1<br>2	Cannot do<br>Can do with support<br>Can do independently	
59	Establishes the trust for patients, families and care team members	0<br>1<br>2	0. Cannot do<br>1. Can do with support<br>2. Can do independently	
60	spend necessary time for communicating with patients, families and care team members	0<br>1<br>2	Cannot do<br>Can do with support<br>Can do independently	
61	Listens and answers the considerations and worries of the patients and families	0<br>1<br>2	Cannot do<br>Can do with support<br>Can do independently	
62	Identifies psychological, nursing care needs through facial expressions and body language of patients	0<br>1<br>2	Cannot do<br>Can do with support<br>Can do independently	
63	Communicates effectively with individuals, families, communities who have communication problems due to disease, psychological problems.	0<br>1<br>2	Cannot do<br>Can do with support<br>Can do independently	
64	Expresses words, behaviors that motivate, encourage patients to be assured of treatment	0<br>1<br>2	Cannot do<br>Can do with support<br>Can do independently	
65	Demonstrates and understands of culture, belief in communication with patients, families and communities	0<br>1<br>2	Cannot do<br>Can do with support<br>Can do independently	
66	Utilizes available audio-visual facilities to support communication with patients, families and communities	0<br>1<br>2	Cannot do<br>Can do with support<br>Can do independently	

Qestionaire for new nurses before training

67	Utilizes effective and proper measures and communication forms to patients and families	0<br>1<br>2	Cannot do<br>Can do with support<br>Can do independently	
68	Identifies the information which need to inform to patients and families	0<br>1<br>2	Cannot do<br>Can do with support<br>Can do independently	
69	Performs psychological preparation for patients and families before informing "bad news"	0<br>1<br>2	Cannot do<br>Can do with support<br>Can do independently	
70	Collects and analyzes information on the needs of health of individuals, families, and communities	0<br>1<br>2	Cannot do<br>Can do with support<br>Can do independently	
71	Determines needs and contents which are necessary to provide health education to individuals, families, and communities	0<br>1<br>2	Cannot do<br>Can do with support<br>Can do independently	
72	Develops health education plan which is suitable to cultural and religious aspects for individuals, families and communities	0<br>1<br>2	Cannot do<br>Can do with support<br>Can do independently	
73	Develops education documents which is suitable to clients' qualifications	0<br>1<br>2	Cannot do<br>Can do with support<br>Can do independently	
74	Performs health consultation, education properly and effectively	0<br>1<br>2	Cannot do<br>Can do with support<br>Can do independently	
75	Evaluate health education result and revised the health education plan based on target and expected results	0<br>1<br>2	Cannot do<br>Can do with support<br>Can do independently	
76	Well maintains the relationship with other team members, considers patient as a partner in the team	0<br>1<br>2	Cannot do<br>Can do with support<br>Can do independently	
77	Well collaborates with other team members to make relevant decisions to improve health care quality	0<br>1<br>2	Cannot do<br>Can do with support<br>Can do independently	
78	Well collaborate with other team members to monitor, take care, treat the patients and complete assigned duties	0<br>1<br>2	Cannot do<br>Can do with support<br>Can do independently	
79	Respects roles and opinions of colleagues	0<br>1<br>2	Cannot do<br>Can do with support<br>Can do independently	
80	Effectively shares information with other care team members	0<br>1<br>2	Cannot do<br>Can do with support<br>Can do independently	
81	Acts as liaison or advocate of the patients to ensure patients' benefits, rights for the patient's safe	0<br>1<br>2	Cannot do<br>Can do with support<br>Can do independently	
82	Performs the principles on patient's records in regards of MOH regulations	0<br>1<br>2	Cannot do<br>Can do with support<br>Can do independently	
83	Maintains the patients records and care sheet in confident and privacy.	0<br>1<br>2	Cannot do<br>Can do with support<br>Can do independently	
84	Records nursing documents to ensure the objectiveness, accuracy, fullness and timely	0<br>1<br>2	Cannot do<br>Can do with support<br>Can do independently	
85	Uses collected data of patients' health conditions for patient's monitoring and care basis.	0<br>1<br>2	Cannot do<br>Can do with support<br>Can do independently	
86	Manages individual's tasks, time effectively and scientifically	0<br>1<br>2	Cannot do<br>Can do with support<br>Can do independently	
87	Identifies tasks or duties that need to be completed based on priorities	0<br>1<br>2	Cannot do<br>Can do with support<br>Can do independently	
88	Organizes, coordinates, assigns and authorizes duties to team members scientifically, properly and effectively	0<br>1<br>2	Cannot do<br>Can do with support<br>Can do independently	
89	Demonstrates understanding of the relationship between management and utilization of resources effectively to ensure quality and safe care for patients	0<br>1<br>2	Cannot do<br>Can do with support<br>Can do independently	
90	use IT in patient management, care and updating professional knowledge	0<br>1<br>2	Cannot do<br>Can do with support<br>Can do independently	
91	Establishes mechanism to manage and maximize operational functions of equipment used for patients care and treatment	0<br>1<br>2	Cannot do<br>Can do with support<br>Can do independently	
92	Develops and perform equipment maintenance plans	0<br>1<br>2	Cannot do<br>Can do with support<br>Can do independently	

93	operates equipment used for patient care to ensure safe and effectiveness and prevent from health care related infection	0<br>1<br>2	Cannot do<br>Can do with support<br>Can do independently	
94	Identifies the cost-effectiveness in the utilization of available resources properly and effectively	0<br>1<br>2	Cannot do<br>Can do with support<br>Can do independently	
95	Develops and implements plans using considering resources effectively within assigned duty	0<br>1<br>2	Cannot do<br>Can do with support<br>Can do independently	
96	Complies with standards and principles of working safety	0<br>1<br>2	Cannot do<br>Can do with support<br>Can do independently	
97	Complies with policies, procedures of infection control prevention	0<br>1<br>2	Cannot do<br>Can do with support<br>Can do independently	
98	Complies with regulations on caring environment control (noise, air, water…)	0<br>1<br>2	Cannot do<br>Can do with support<br>Can do independently	
99	Complies with regulations on waste management and treatment	0<br>1<br>2	Cannot do<br>Can do with support<br>Can do independently	
100	Complies with steps of fire protection, earthquake or other urgent cases	0<br>1<br>2	Cannot do<br>Can do with support<br>Can do independently	
101	Demonstrates the understandings of professional health related matters and laws on working safety	0<br>1<br>2	Cannot do<br>Can do with support<br>Can do independently	
102	Be aware of the necessity of quality assurance activities, quality improvement through research, feedback and evaluation of regular practice	0<br>1<br>2	Cannot do<br>Can do with support<br>Can do independently	
103	Detects and reports environmental risks in patient care and make appropriate corrective action	0<br>1<br>2	Cannot do<br>Can do with support<br>Can do independently	
104	Receives feedbacks from patients, families and relevant people to improve quality of caring activities	0<br>1<br>2	Cannot do<br>Can do with support<br>Can do independently	
105	Applies proper quality improvement measures	0<br>1<br>2	Cannot do<br>Can do with support<br>Can do independently	
106	Participates in quality improvement activities at the facility	0<br>1<br>2	Cannot do<br>Can do with support<br>Can do independently	
107	Shares information relating to patient’s health conditions to other team members	0<br>1<br>2	Cannot do<br>Can do with support<br>Can do independently	
108	Discusses on caring sheet to improve and overcome the issues relating to expertise and administrative procedures	0<br>1<br>2	Cannot do<br>Can do with support<br>Can do independently	
109	Gives proper proposals of care measures and disease prevention	0<br>1<br>2	Cannot do<br>Can do with support<br>Can do independently	
110	Uses evidence-based application on care practice to enhance safe in patient care	0<br>1<br>2	Cannot do<br>Can do with support<br>Can do independently	
111	Identifies and selects research issues and areas properly, necessity and feasibly	0<br>1<br>2	Cannot do<br>Can do with support<br>Can do independently	
112	Applies proper measures to perform selected researches	0<br>1<br>2	Cannot do<br>Can do with support<br>Can do independently	
113	Uses proper statistical methods to analyze and explain the collected data	0<br>1<br>2	Cannot do<br>Can do with support<br>Can do independently	
114	Proposes suitable solutions based on researching results	0<br>1<br>2	Cannot do<br>Can do with support<br>Can do independently	
115	Presents, shares researching results, experiences with colleagues, patients and relevant people	0<br>1<br>2	Cannot do<br>Can do with support<br>Can do independently	
116	Utilizes researching results on nursing practice. Uses evidence from scientific researches to improve nursing care practice	0<br>1<br>2	Cannot do<br>Can do with support<br>Can do independently	
117	Clearly identifies targets, professional development expectation, own learning needs, strengths, weaknesses	0<br>1<br>2	Cannot do<br>Can do with support<br>Can do independently	
118	Continuous leaning in order to update knowledge, skills and utilizes learned knowledge to improve nursing care practice quality	0<br>1<br>2	Cannot do<br>Can do with support<br>Can do independently	

119	Joins in activities of professional organizations	0<br>1<br>2	Cannot do<br>Can do with support<br>Can do independently	
120	Promotes image of nurse, demonstrates good behavior and character, appropriate attire, convincing words and proper manners	0<br>1<br>2	Cannot do<br>Can do with support<br>Can do independently	
121	Possesses positive attitude towards change and criticism, listens to suggestions and recommendations, tries new methods and adapts to changes willingly	0<br>1<br>2	Cannot do<br>Can do with support<br>Can do independently	
122	Performs cares according to nursing care standards	0<br>1<br>2	Cannot do<br>Can do with support<br>Can do independently	
123	contribute to enhance the career qualification and development for colleagues	0<br>1<br>2	Cannot do<br>Can do with support<br>Can do independently	
124	Contributes to improve nurse's roles, positions, nursing industry in medial field and society	0<br>1<br>2	Cannot do<br>Can do with support<br>Can do independently	
125	Practice in accordance with health-related laws, regulations of MOH and nursing practice	0<br>1<br>2	Cannot do<br>Can do with support<br>Can do independently	
126	Complies with regulations at the working place	0<br>1<br>2	Cannot do<br>Can do with support<br>Can do independently	
127	Well performs code of treatment of the facility/organization and laws	0<br>1<br>2	Cannot do<br>Can do with support<br>Can do independently	
128	records and stores caring profiles and documents relating to patients, patient's health issues in line with care practice standards.	0<br>1<br>2	Cannot do<br>Can do with support<br>Can do independently	
129	Takes personal responsibility in making care decisions and interventions.	0<br>1<br>2	Cannot do<br>Can do with support<br>Can do independently	
130	Complies to the code of ethics for nurses in nursing practice.	0<br>1<br>2	Cannot do<br>Can do with support<br>Can do independently	
131	Reports unethical and immoral incidents to competent authorities and be responsible for that report.	0<br>1<br>2	Cannot do<br>Can do with support<br>Can do independently	

COMPLETE
